# Enlightening the black and white: species delimitation and UNITE species hypothesis testing in the *Russula albonigra* species complex

**DOI:** 10.1186/s43008-021-00064-0

**Published:** 2021-08-02

**Authors:** Ruben De Lange, Slavomír Adamčík, Katarína Adamčíkova, Pieter Asselman, Jan Borovička, Lynn Delgat, Felix Hampe, Annemieke Verbeken

**Affiliations:** 1 Research Group Mycology, Department of Biology, Ghent University, K.L. Ledeganckstraat 35, 9000 Ghent, Belgium; 2 Institute of Botany, Plant Science and Biodiversity Center, Slovak Academy of Sciences, Dúbravská cesta 9, 845 23 Bratislava, Slovakia; 3 Institute of Forest Ecology Slovak Academy of Sciences, Akademická 2, 949 01 Nitra, Slovakia; 4 Institute of Geology of the Czech Academy of Sciences, Rozvojová 269, 165 00 Prague 6, Czech Republic; 5 Nuclear Physics Institute of the Czech Academy of Sciences, Hlavní 130, 250 68 Husinec-Řež, Czech Republic; 6 Meise Botanic Garden, Research Department, Nieuwelaan 38, 1860 Meise, Belgium

**Keywords:** Basidiomycota, Coalescent species delimitation, Ectomycorrhizal fungi, New species, Phylogeny, *Russulaceae*, *Russulales*, *Russula* subgen. *Compactae*, Integrative taxonomy, Typification, New taxa

## Abstract

*Russula albonigra* is considered a well-known species, morphologically delimited by the context of the basidiomata blackening without intermediate reddening, and the menthol-cooling taste of the lamellae. It is supposed to have a broad ecological range and a large distribution area. A thorough molecular analysis based on four nuclear markers (ITS, LSU, RPB2 and TEF1-α) shows this traditional concept of *R*. *albonigra s. lat.* represents a species complex consisting of at least five European, three North American, and one Chinese species. Morphological study shows traditional characters used to delimit *R*. *albonigra* are not always reliable. Therefore, a new delimitation of the *R*. *albonigra* complex is proposed and a key to the described European species of *R*. subgen. *Compactae* is presented. A lectotype and an epitype are designated for *R*. *albonigra* and three new European species are described: *R*. *ambusta*, *R*. *nigrifacta,* and *R*. *ustulata*. Different thresholds of UNITE species hypotheses were tested against the taxonomic data. The distance threshold of 0.5% gives a perfect match to the phylogenetically defined species within the *R. albonigra* complex. Publicly available sequence data can contribute to species delimitation and increase our knowledge on ecology and distribution, but the pitfalls are short and low quality sequences.

**Supplementary Information:**

The online version contains supplementary material available at 10.1186/s43008-021-00064-0.

## INTRODUCTION

Molecular identification of species gained importance over the last decades (Matute and Sepulveda 2019). As new techniques became available and more easily accessible, the number of publications using sequence data increased immensely (Hibbett et al. 2011; Kõljalg et al. 2013; Nilsson et al. 2018). The main problems with molecular identification are poor taxon coverage and misidentifications in many public sequence databases, as well as high infraspecific variability of DNA regions causing poor performance of the barcoding gap (Kõljalg et al. 2005; Badotti et al. 2017; Hofstetter et al. 2019). To overcome some of these problems, the UNITE database was created (https://unite.ut.ee/). UNITE targets the still most widely used, universal fungal barcode: the nuclear internal transcribed spacer (ITS) region to provide high-quality reference records (Nilsson et al. 2018). UNITE groups individual ITS sequences into species hypotheses (SHs) at several distance thresholds (i.e. between 0 and 3%), each assigned a unique digital object identifier (DOI) which allows unambiguous reference across studies. These species hypotheses are either assigned automatically with a representative sequence or manually by a taxonomic expert with a reference sequence (Kõljalg et al. 2013; Nilsson et al. 2018).

Most *Basidiomycota*, the second largest phylum of fungi, are *Agaricomycotina* (Naranjo-Ortiz and Gabaldon 2019). A considerable part of this subphylum’s diversity is concentrated in large genera of ectomycorrhiza-forming agarics, e.g. *Cortinarius* and *Russula*, which exhibit very different evolutionary rates (Ryberg and Matheny 2012; Varga et al. 2019). Recent studies on lineages of closely related *Russula* members revealed that they often comprise closely related species diversified by ecological adaptation and isolation by distance or disjunction (Adamčík et al. 2016b; Caboň et al. 2019; Looney et al. 2020). This makes the recognising of these species relevant, despite their similarity in the ITS barcode. The efforts to barcode fungal species sometimes fail, due to lack of taxonomic studies addressing the species concept based on type collections (Kõljalg et al. 2020). Our current studies on *Russula* subgen. *Compactae* recognised *Russula albonigra* as one such example. Its morphological concept is historically established, and the species is traditionally recognised by the moderately distant, relatively narrow lamellae, the menthol-cooling taste of the lamellae, and the surface of the cap and the stipe, as well as the lamellae, that are strongly and rapidly blackening (hence, resulting in black-and-white contrast of bruised and untouched parts). Most publications report blackening without intermediate reddening of the context and surface (Romagnesi 1967; Adamčík and Buyck 2014). Microscopically, the species is defined by a low reticulate spore ornamentation and pileocystidia without apical knobs (Romagnesi 1967). However, our current search in two databases, which seek to adopt a nomenclatural concept for fungal operational units defined by the ITS barcode, recovered inconsistencies. Samples identified as *R. albonigra* in the BOLD database (https://www.boldsystems.org/) do not match any of the species hypothesis under this species name published in UNITE (https://unite.ut.ee/), and vice versa.

In this study, we use sequence data of four DNA markers and a detailed morphological revision to test the taxonomic status within the *R. albonigra* complex. To test different distance thresholds of UNITE species hypotheses, we used the strict genealogical concordance assessing the extent of genetic concordance across loci, the coalescent based species delimitation modelling the genealogical history of individuals back to a common ancestor and morphological differences.

## MATERIAL AND METHODS

### Sampling

This study is based on collections from sampling trips in Belgium (2016, 2017 and 2018), Italy (1997, 2000 and 2016), Norway (2016), Slovakia (2003, 2006, 2008, 2009, 2011, 2015 and 2017), and Sweden (2016 and 2018). All collections are deposited in the Herbarium Universitatis Gandavensis (GENT) or the Slovak Academy of Sciences (SAV). Supplementary collections were requested from the Mycological Department of the National Museum in Prague, Czech Republic (PRM), and from the personal collections of Felix Hampe, Jesko Kleine, and Helga Marxmüller (the latter recently deposited in the State Museum of Natural History Karlsruhe, KR).

Samples that could belong in the *Russula albonigra* complex were selected based on morphology: (1) collections that were identified as *R*. *albonigra* in the field based on macro-morphology; and (2) fungarium collections labelled as *R*. *albonigra* based on both macro- and micro-morphological observations. The selected samples were molecularly checked using the ITS marker as a guideline and not included for further study when not placed within the *R*. *albonigra* complex.

### Morphological analysis

The macroscopic description is based on observations from fresh material, with colour codes following Kornerup and Wanscher (1978), guaiac reactions referring to Chalange (2014), and spore print colour codes following the scale of Romagnesi (1967). The microscopic description and terminology follow Adamčík et al. (2019). Microscopic characters were studied from dried material, spores were observed in Melzer’s reagent, elements of the hymenium and pileipellis were observed in Congo red in L4 after ca. 10 s in KOH 10%. Basidiospores were measured using a crosshair eyepiece on a Zeiss Axioskop 2 microscope. Line drawings of spores were made based on stacked photographs (Nikon Eclipse Ni-U microscope, stacking software: Extended Depth of Field, Nikon Nis Elements module) at an original magnification of 5000×. Measurements of other elements were made using an eyepiece micrometer and line drawings were prepared with the aid of a camera lucida (Olympus U-DA) on an Olympus CX21 or BX43 microscope, at original magnifications of 1500× or 2000×. Tissues were mounted in Cresyl Blue ([Bibr CR9][Bibr CR9]), sulfovanillin (Cabon et al. 2017) and treated with carbolfuchsin (Romagnesi 1967) to observe the presence and colour changes of incrustations and cystidium contents. All cited collections in the species descriptions have been sequenced, at least for ITS.

The key provided in the Taxonomy section is based on our observations of species within the *R*. *albonigra* complex, and following the traditional species concepts in literature for the other taxa. *Russula clementinae* is not included in the key as it is interpreted as a synonym of *R*. *densifolia* (Sarnari 1998).

### Molecular analysis

DNA extraction and amplification was performed in either the molecular laboratory of Ghent University or that of the Slovak Academy of Sciences. Sequencing of PRM collections was conducted within the study of Leonhardt et al. (2019).

In Ghent University, DNA from fresh material was extracted using the CTAB extraction described in Nuytinck and Verbeken (2003). DNA from dried material was extracted using a modified CTAB protocol (Tel-Zur et al. 1999; modified by Meise Botanic Garden and Research Group Mycology of Ghent University). The original protocol was optimized for cacti species. These plant types tend to have larger cells compared to fungi. Hence, the ratio DNA content:biomass for fungi is much higher. Therefore, less biomass is needed as starting material and buffer volumes were adjusted consequently. Because mucilaginous polysaccharides have not been observed in previous Fungal DNA extractions the use of sorbitol in our extractions was omitted. Additionally, the use of CTAB as detergent to brake open fungal cells seemed to suffice to access the fungal DNA in an efficient way. Hence, the extra sarkosyl added in the lysis step was also omitted from our protocol. Protocols for PCR amplification follow Le et al. (2007). In the Slovak Academy of Sciences, total genomic DNA was extracted from dried material using the EZNA Fungal DNA Mini Kit (Omega Bio-Tek, Norcross, GA, USA) following the manufacturer’s instruction. Amplification of DNA was performed in a PCR reaction mix consisting of approximately 2 ng/μl of template DNA, forward and reverse primers (10 pmol/μl), 5× HOT FIREPol® Blend Master Mix (Solis BioDyne, Tartu, Estonia) and molecular grade water added up to 20 μl. Four nuclear markers were amplified: (1) the internal transcribed spacer region of ribosomal DNA (ITS), comprising the ITS1 and ITS2 spacer regions and the ribosomal gene 5.8S, using primers ITS1-F and ITS4 (White et al. 1990; Gardes and Bruns 1993); (2) a part of the ribosomal large subunit 28S region (LSU), using primers LR0R and LR5 (Moncalvo et al. 2000); (3) the region between the conserved domains 6 and 7 of the second largest subunit of the RNA polymerase II (RPB2), using primers bRPB2-6F and fRPB2–7cR or bRPB2–7.1R (Liu et al. 1999; Matheny 2005); and (4) the translation elongation factor 1-alpha (TEF1α), using primer pairs EF1-1018F and EF1-1620R or tef1F and tef1R (Morehouse et al. 2003; Stielow et al. 2015). PCR products from Ghent University were sequenced using an automated ABI 3730 XL capillary sequencer at Macrogen. In the Slovak Academy of Sciences, the PCR products were purified using Qiaquick PCR Purification Kit (Qiagen, Hilden, Germany) and samples were sequenced by the Seqme company (Dobříš, Czech Republic).

Forward and reverse sequences were assembled into contigs and edited where needed with BioloMICS (BioAware SA NV). All sequences generated were deposited in GenBank (Table 1).

**Table 1:** Specimens and GenBank accession numbers of DNA sequences used in the multi-locus phylogenetic analysis

Taxon	Voucher collection (herbarium)	Country	ITS	LSU	RPB2	TEF1α
*Russula acrifolia*	LD 16–022 (GENT)	Sweden	MW172319	MW182479	MW306683	MW273325
*Russula acrifolia*	RDL 18–012 (GENT)	Sweden	MW172320	MW182480	MW306684	MW273326
*Russula acrifolia*	RDL 18–021 (GENT)	Sweden	MW172321	MW182481		MW273327
*Russula adusta*	LD 16–025 (GENT)	Sweden	MW172316		MW306682	MW273322
*Russula adusta*	RDL 18–020 (GENT)	Sweden	MW172317	MW182477		MW273323
*Russula adusta*	RDL 18–028 (GENT)	Sweden	MW172318	MW182478		MW273324
*Russula albonigra*	JK RUS 13090603 (Jesko Kleine*)	Germany	MW172296	MW182461	MW306670	
*Russula albonigra*	SAV F-755 (SAV)	Slovakia	MW172291			
*Russula albonigra*	SAV F-2559 (SAV)	Slovakia	MW172292			
*Russula albonigra*	SAV F-20177 (SAV)	Slovakia	MW172298	MW182463	MW306672	MW273311
***Russula albonigra***	**SAV F-20197 (SAV)**	Slovakia	MW172299	MW182464	MW306673	MW273312
*Russula albonigra*	SAV F-3465 (SAV)	Slovakia	MW172293	MW182460	MW306669	MW273309
*Russula albonigra*	SAV F-4776 (SAV)	Slovakia	MW172297	MW182462	MW306671	MW273310
*Russula albonigra*	PRM 934322 (PRM)	Czech Republic	MW172294			
*Russula albonigra*	PRM 924409 (PRM)	Czech Republic	MW172295			
*Russula ambusta*	FH 2008 ST01 (Felix Hampe*)	Germany	MW172300	MW182465		
***Russula ambusta***	**SAV F-3358 (SAV)**	Slovakia	MW172301	MW182466		
*Russula* cf. *anthracina*	RDL 16–031 (GENT)	Italy	MW172313	MW182474	MW306679	MW273319
*Russula* cf. *anthracina*	RDL 16–058 (GENT)	Italy	MW172314	MW182475	MW306680	MW273320
*Russula* cf. *anthracina*	RDL 18–026 (GENT)	Sweden	MW172315	MW182476	MW306681	MW273321
*Russula archaeosuberis*	BB 12.085 (PC)	Italy	KY800355°	KU237593°	KU237878°	KU238019°
*Russula atramentosa*	FH 2011-002R (Felix Hampe*)	Belgium	MW172322	MW182482	MW306685	MW273328
*Russula atramentosa*	RDL 16–050 (GENT)	Italy	MW172323		MW306686	
*Russula atramentosa*	FH0170824–02 (Felix Hampe*)	Germany	MW172324	MW182483	MW306687	MW273329
*Russula camarophylla*	MPG11–7-09 (PC)	Spain	KY800353°	KU237579°	KU237865°	KU238006°
*Russula cantharellicola*	UC1999420	United States	KF306036°			
*Russula cascadensis*	OSC 1064009 (OSC)	United States	EU526006°			
***Russula cortinarioides***	**BB 07.133 (PC)**	United States	KP033485°		KP033507°	
*Russula cortinarioides*	BB 07.103 (PC)	United States	KP033480°	KP033491°	KP033502°	KU237985°
*Russula densifolia*	RDL 16–001/2 (GENT)	Belgium	MW172325	MW182484	MW306688	MW273330
*Russula densifolia*	RDL 18–052 (GENT)	Belgium	MW172326	MW182485	MW306689	MW273331
*Russula densifolia*	RDL 17–024 (GENT)	Belgium	MW172327	MW182486	MW306690	MW273332
*Russula densissima*	FH 2014 ST04 (Felix Hampe*)	Germany	MW172328		MW306691	
*Russula densissima*	FH 2010 ST02 (Felix Hampe*)	Germany	MW172329		MW306692	
*Russula dissimulans*	BPL704 (TENN)	United States	KY848513°			
*Russula earlei*	BPL245 (TENN)	United States	KT933961°	KT933820°	KT933891°	
*Russula* cf. *eccentrica*	BB 07.044 (PC)	United States	KP033479°	KP033490°	KP033501°	KU237937°
*Russula* cf. *eccentrica*	BB 07.132 (PC)	United States	KP033478°	KP033489°	KP033500°	KU237926°
*Russula* cf. *fuliginosa*	FH RUS 14091001 (Felix Hampe*)	Slovakia	MW172330	MW182487	MW306693	MW273333
*Russula* cf. *fuliginosa*	FH RUS 14091201 (Felix Hampe*)	Slovakia	MW172331	MW182488	MW306694	MW273334
*Russula gossypina*	BB 06.002 (PC)	Madagascar	KY800350°	KU237450°	KU237736°	KU237886°
*Russula ingwa*	MEL2101936	Australia	EU019919°			
***Russula khanchanjungae***	**AV KD KVP 09–106 (GENT)**	India	KR364129°	JN389004°	JN375607°	
*Russula lateriticola*	BB 06.031 (PC)	Madagascar	KP033476°	KP033487°	KP033498°	KU237888°
*Russula nigricans*	RDL 17–004 (GENT)	Belgium	MW172332	MW182489	MW306695	MW273335
*Russula nigricans*	RDL 17–007 (GENT)	Belgium	MW172334	MW182491	MW306697	MW273337
*Russula* cf. *nigricans*	RDL 17–005 (GENT)	Belgium	MW172333	MW182490	MW306696	MW273336
*Russula nigrifacta*	RDL 16–028 (GENT)	Italy	MW172307		MW306676	MW273316
***Russula nigrifacta***	**RDL 16–044 (GENT)**	Italy	MW172308	MW182470	MW306677	MW273317
*Russula nigrifacta*	RDL 16–063 (GENT)	Italy	MW172306			MW273315
*Russula nigrifacta*	SAV F-2418 (SAV)	Slovakia	MW172304			
*Russula nigrifacta*	SAV F-2419 (SAV)	Slovakia	MW172303	MW182468		
*Russula nigrifacta*	SAV F-1501 (SAV)	Slovakia	MW172302	MW182467	MW306674	MW273314
*Russula nigrifacta*	SAV F-3006 (SAV)	Slovakia	MW172305	MW182469	MW306675	
*Russula polyphylla*	BB 07.134 (PC)	United States	KP033486°	KP033497°	KP033508°	KU238023°
*Russula polyphylla*	BB 07.023 (PC)	United States	KP033481°	KP033492°	KP033503°	KU237986°
*Russula roseonigra*	KR-M-0042973/MxM R-9308 (KR)	France	MW172335			
*Russula roseonigra*	FH 2014 ST01 (Felix Hampe*)	Germany	MW172336		MW306698	MW273338
*Russula roseonigra*	RDL 16–024 (GENT)	Italy	MW172337	MW182492	MW306699	MW273339
*Russula* sp.	FH 12–064 (GENT)	Thailand	MN130076°		MN380517°	
*Russula* sp. 1	RW 1975 (GENT)	Italy	MW172309	MW182471		
***Russula ustulata***	**AV 16–019 (GENT)**	Norway	MW172312	MW182473	MW306678	MW273318
*Russula ustulata*	SAV F-2610 (SAV)	Italy	MW172310	MW182472		
*Russula ustulata*	PRM 924452 (PRM)	Czech Republic	MW172311			

In bold: types; * personal herbarium; ° sequences not generated in this study

#### Phylogenetic analysis

Identifications of publicly available sequences of fungi often match contrasting taxonomic species concepts. To provide reliable sampling in accordance with traditional species concepts we selected three representative collections of each European species described within *R*. subgen. *Compactae*, identified using the most recent and reliable keys (Romagnesi 1967; Sarnari 1998). These collections, together with the collections of *R. albonigra s. lat.* were sequenced by us (Table 1). For non-European species, sequences used in Adamčík et al. (2019) or Buyck et al. (2020) were included if multiple of the markers used in this study were available for these samples, with an ITS sequence obligatory. Four species of *Russula* subgen. *Archaeae* were used as an outgroup, because the recent phylogenies of the genus place this subgenus as sister to *R*. subgen. *Compactae*.

**Fig. 1:**
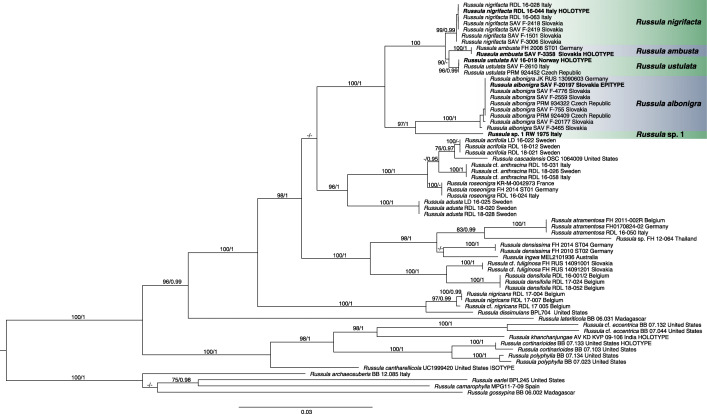
Maximum Likelihood (ML) tree of *Russula* subgen. *Compactae*, based on concatenated ITS, LSU, RPB2 and TEF1α sequence data. ML bootstrap values > 75 and BI posterior probabilities > 0.95 are shown

Sequences were aligned using the online version of the multiple sequence alignment program MAFFT v7 (Katoh and Toh 2008), using the E-INS-i strategy. Trailing ends of the alignments were trimmed and the alignments were manually edited when necessary in MEGA7 (Kumar et al. 2016). The alignments can be obtained from the first author and TreeBASE (Submission ID 26815). The alignments were partitioned into following partitions: ITS-LSU-alignment: partial 18S, ITS1, 5.8S, ITS2, LSU; RPB2-alignment: the RPB2 intron and the first, second and third codon positions of the exon; TEF1α-alignment: the first and second intron and the first, second and third codon positions. PartitionFinder2 was used to find the appropriate partitioning scheme and substitution models using the Akaike information criterion (AICc) with a greedy search over all models (Guindon et al. 2010; Lanfear et al. 2012; Lanfear et al. 2017). Maximum likelihood (ML) analyses were conducted with IQ-Tree (Nguyen et al. 2014; Chernomor et al. 2016) using standard bootstrapping analysis (1000 replicates). Bayesian inference (BI) was executed with MrBayes v3.2.6 (Huelsenbeck and Ronquist 2001; Ronquist and Huelsenbeck 2003). Two independent parallel runs of one cold and three heated chains were run for ten million (single-locus datasets) or twenty million generations (multi-locus dataset) with a sample frequency of 100. Potential Scale Reduction Factor (PSRF) values approached 1.0. Convergence and Effective Sample Size (ESS) statistics of the runs were also examined with Tracer v1.7.1 (Rambaut et al. 2018). A burn-in sample of 20% was excluded before constructing the majority rule consensus tree. Analyses were first performed on each alignment separately and visually checked for incongruence. Significant incongruence was assumed if two different relationships (one monophyletic and the other non-monophyletic) for any set of taxa were supported with bootstrap values (BS) ≥ 70 or posterior probabilities (PP) ≥ 90. The resulting gene trees did not show any supported conflicts, therefore all alignments could be concatenated. The concatenated alignment was used for the multi-locus phylogenetic analyses (Fig. 1).

#### Coalescent species delimitation approaches

For species delimitation under the multispecies coalescent model a part of the alignment used in the multi-locus phylogenetic analyses mentioned above, comprising members of the *Russula albonigra* complex, was used. A total of five potential species units (as proposed by the ML and BI trees) were evaluated as the full model. Two coalescent species delimitation methods were performed to test these species hypotheses. The first method was implemented in Bayesian Phylogenetics and Phylogeography, BP&P v4.3.8 (Yang 2015). We performed analysis A11 (Yang and Rannala 2014) for unguided species delimitation using rjMCMC algorithm 0 (Yang and Rannala 2010). Analyses were run with several values for the fine-tune parameter (ε = 2, 5, 10 and 20) and we assigned equal probabilities to the rooted species trees as a species model prior. Because the prior distributions on the ancestral population size and root age can affect the posterior probabilities of the model, we considered three different combinations of priors (based on the idea of Leache and Fujita (2010)): (1) θ ~ IG(3,0.002) and τ ~ IG(3,0.002), (2) θ ~ IG(3,0.02) and τ ~ IG(3,0.02), and (3) θ ~ IG(3,0.02) and τ ~ IG(3,0.002) (with α = 3 for a diffuse prior as proposed in the manual). For each combination of settings the analysis was run twice with a different seed (to confirm consistency between runs) for 200,000 generations (sampling interval of two) and a burn-in of 50,000. As a second species delimitation method we used the STACEY v1.2.5 (Jones 2017) package implemented in BEAST2 (Bouckaert et al. 2019). The xml-file for the BEAST2 runs were prepared in BEAUTi v2.6.3 (Drummond et al. 2012). We used following partitions: for the nrDNA (1) 5.8S, (2) ITS1 + ITS2 and (3) LSU; for the protein coding loci the introns and the first, second and third codon positions of the exons. PartitionFinder2 was used to find the appropriate substitution models. The substitution rate of each partition was estimated independently of the others. Clock and tree model parameters were estimated independently for the nrDNA and each protein coding locus. We used a lognormal, relaxed clock model and a Yule tree model. The Collapse Height parameter ε was set to 10^− 5^. The Collapse Weight parameter ω was estimated and given a uniform prior on [0,1] so that every number of species between five and one is regarded as equally likely a priori. We ran five parallel MCMC runs for one billion generations sampling every 1000th tree. Convergence and Effective Sample Size (ESS) statistics of the runs were examined with Tracer v1.7.1. Twenty percent of each run was discarded as burn-in and the remaining posterior samples were combined using LogCombiner v2.6.3 (Drummond and Rambaut 2007) and used to calculate the most likely number of clusters (i.e., putative species), using SpeciesDelimitationAnalyzer (Jones et al. 2014).

#### Species hypothesis and threshold testing

**Fig. 2:**
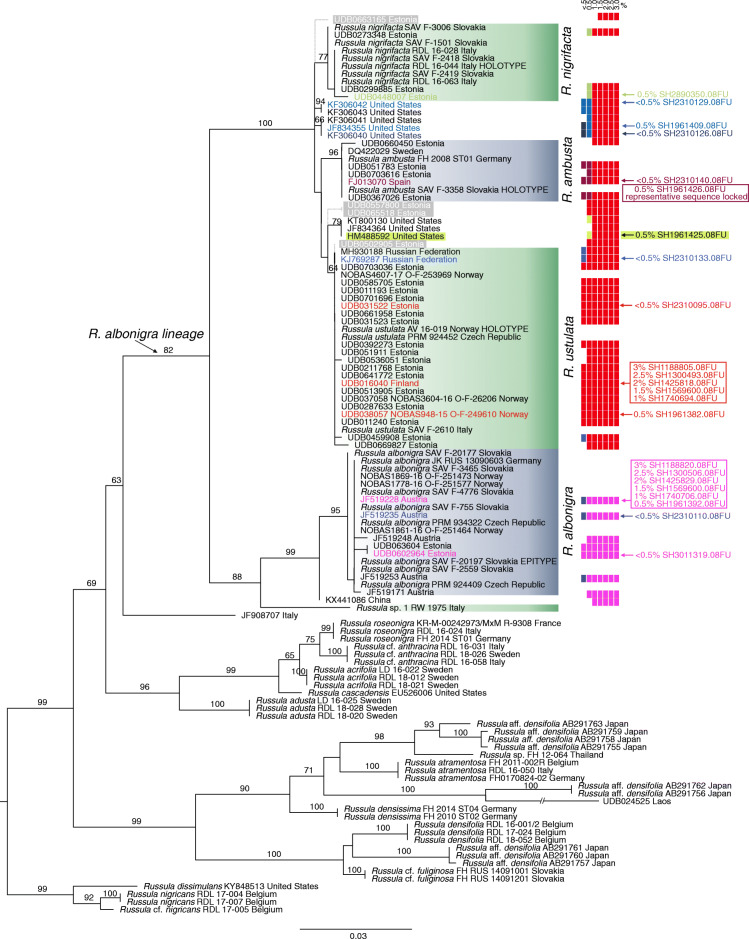
Maximum Likelihood (ML) tree of *Russula* sect. *Nigricantinae*, based on ITS sequence data. ML bootstrap values > 60 are shown. Colour bars of similar colour within each column represent individual UNITE species hypotheses. Missing bars represent unresolved clustering. Samples in colour are representative sequences labelled with SH numbers. Grey shaded samples in white are plotted to the tree based on similarities in distinguishing nucleotide positions (uncertain positions are labelled by grey dotted lines)

ITS sequences generated by authors of this study and used in the multi-locus phylogeny were combined with all ITS sequences, either labelled as *R*. *albonigra* or showing high similarity (97%) to our sequences of *R. albonigra* *s.lat*., available on UNITE (https://unite.ut.ee/), GenBank (www.ncbi.nlm.nih.gov) and BOLD (https://www.boldsystems.org/) databases. Accession numbers are given in Fig. 2. The clade containing *R. nigricans* and *R. dissimulans* is in a basal position within *R*. sect. *Nigricantinae* (see Fig. 1) and is chosen as the outgroup. Short sequences (containing only ITS1 or ITS2) and sample UDB065518 (containing many ambiguities and differences in conserved domains compared to other sequences of the group) were excluded from the analysis. The sequences were aligned following the same principles as mentioned above. The alignment was partitioned into following partitions: ITS1, 5.8S and ITS2. A ML analysis was conducted with IQ-Tree (Nguyen et al. 2014; Chernomor et al. 2016) using the option to first test for the best substitution model (Kalyaanamoorthy et al. 2017) and standard bootstrapping analysis (1000 replicates). The excluded samples were later plotted on the tree based on similarities in distinguishing nucleotide positions (Additional file 1). We identified the single nucleotide positions distinguishing the species of the *R*. *albonigra* complex and compared the excluded samples to the good quality sequences based on these positions to estimate their placement in the tree. SH inclusiveness across sequence distance threshold values, as it is shown on UNITE, is plotted against the tree.

## RESULTS

### Multi-locus phylogenetic analyses

All 24 sequences generated by the authors of specimens putatively identified as *R. albonigra*, are placed within *R*. subgen. *Compactae* and group together with other members of *R*. sect. *Nigricantinae* (Fig. 1). All but two of these sequences are placed in one strongly supported clade, here further referred to as the *R*. *albonigra* complex. The two sequences outside the *R*. *albonigra* complex are placed in either the *R*. *atramentosa* clade or the *R. anthracina* clade (these sequences were not used for the final analysis and are not shown in the trees). Our molecular analysis shows the presence of five distinct European clusters within the *R*. *albonigra* complex (Fig. 1). They form four well supported clades and one singleton collection on a long branch. The overall topology of the ML and BI tree was congruent.

The name *R*. *albonigra* is assigned to a clade with two collections from the Czech Republic, PRM 924409 and PRM 934322, that originate from the type collecting area. Three species, *R*. *ambusta*, *R*. *nigrifacta* and *R*. *ustulata*, are described here as new. One species is represented by a singleton position in the tree, placed as sister to *R. albonigra* and is labelled as *Russula* sp. 1. These two species form a sister clade to a larger clade containing *R*. *ambusta*, *R*. *nigrifacta,* and *R*. *ustulata*. The relations within the latter clade are not well supported in the BI tree.

### Coalescent-based species delimitation

The full set of proposed species (i.e. five species) was recovered as the highest supported species model in the BP&P analysis under each combination of settings, with posterior probabilities ranging from 0.91 to 0.99. Also the STACEY analysis resulted in the highest probability (posterior probability of 0.99) for five minimal clusters (species). Both coalescent delimitation methods confirmed the species hypothesis for all five clusters in the multi-locus phylogenetic analysis.

### ITS analysis and optimal SH distance

When searching for UNITE species hypotheses labelled as *R. albonigra,* at every threshold, two species hypotheses are found that are not placed within the *R*. *albonigra* complex defined by our multi-locus analysis. Both are represented by a singleton sequence. The first, UDB024525 represents a collection from Lao People’s Democratic Republic and seems more closely related to *R. atramentosa.* The second, JF908707 represents a collection from Italy with an isolated position in the phylogeny (Fig. 2).

The general topology of the ITS tree is congruent with the multi-locus tree and all sequences of the *R. albonigra* complex generated by the authors of this study are again placed within this monophyletic group (Fig. 2). The ITS analyses revealed the presence of three additional North American clusters and one Chinese collection of singleton position, within the *R. albonigra* complex. Two of the North American clusters are supported and probably represent well defined species, while the status of the three sequences from the USA (KF306041, JF834355 and KF306040) is uncertain and requires more sequence data to resolve. The singleton Chinese sample (KX441086) probably represents an undescribed species sister to *R. albonigra.* Furthermore, Fig. 2 shows an overview of the different UNITE species hypotheses within the *R*. *albonigra* complex at different thresholds. At a threshold of 1% or higher two species hypotheses are recognised (for SH numbers see red and pink boxes in the Fig. 2). The first one labelled as *R. albonigra* with the representative UNITE sequence UDB016040 covers *R*. *ambusta*, *R*. *nigrifacta*, *R*. *ustulata* and the North American species. The second one with the representative sequence JF519228 is labelled as *Russula* sp*.* and covers *R*. *albonigra* and the Chinese species. At a threshold of 0.5%, all European species (except for *R*. sp. 1 which is not represented by any public sequence) and two North American species are supported. Threshold < 0.5% gives additional units within the phylogenetic species that were not supported by our multi-locus analysis.

## TAXONOMY

The species within the *Russula albonigra* complex are characterised by the moderately distant, relatively narrow lamellae, the context that is rapidly and strongly blackening, generally without intermediate reddening. In some cases though, some slight reddening is observed. The taste of the lamellae and flesh is never acrid, but can be menthol-cooling. Microscopically, the species are defined by spores with low and dense warts forming subreticulate to reticulate ornamentation, long pileocystidia (if present) and a cystidial content which is not reacting in sulfovanillin.

**Fig. 3:**
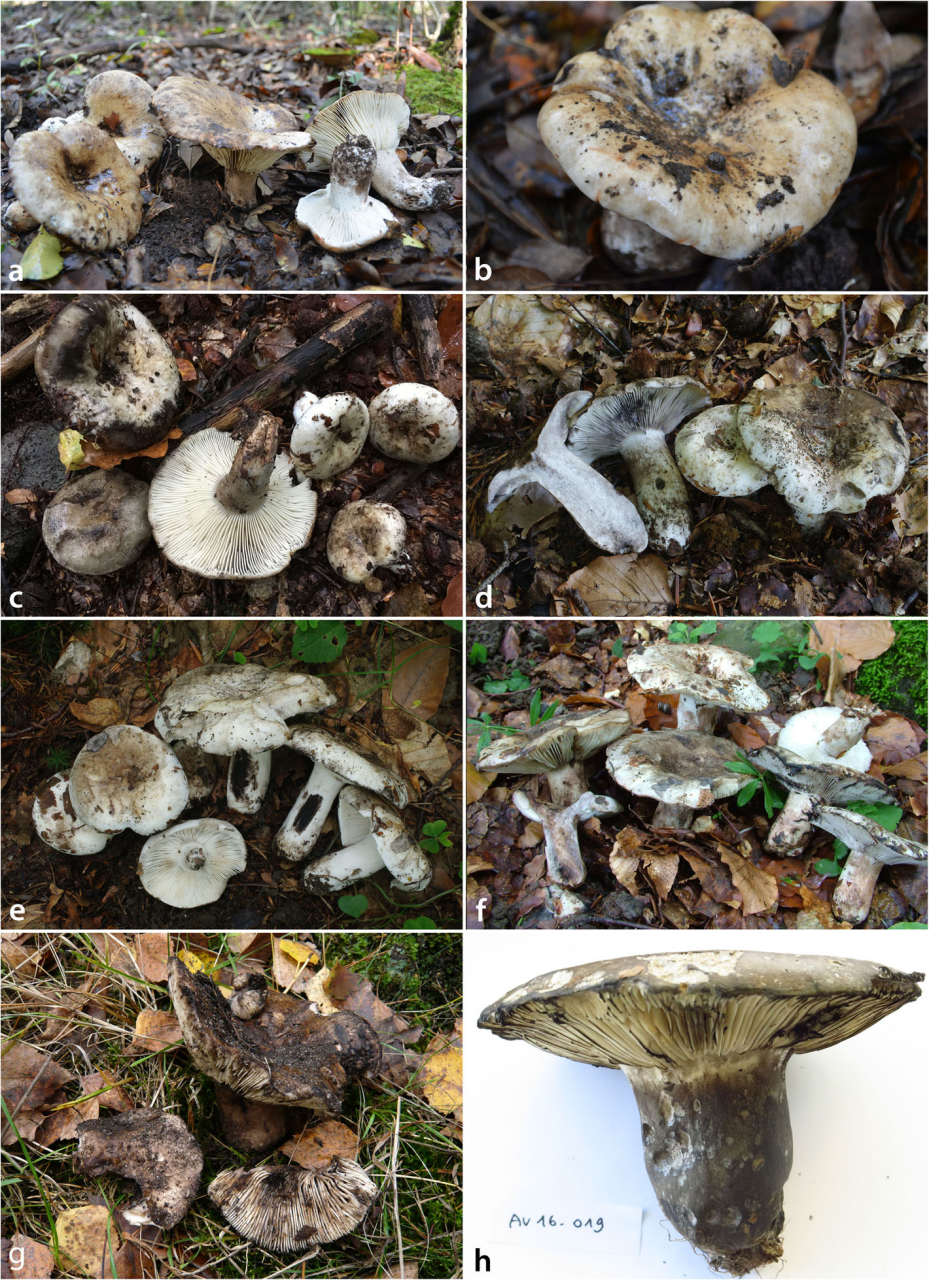
Basidiomata. **a-b** *Russula nigrifacta* (RDL 16–044, holotype). **c-f** *Russula albonigra* (c: SAV F-20197, epitype; **d** PRM 934322; **e** PRM 924409; **f** SAV F-20177). **g** *Russula ambusta* (FH 2008 ST01). **h** *Russula ustulata* (AV 16–019, holotype)

**Fig. 4:**
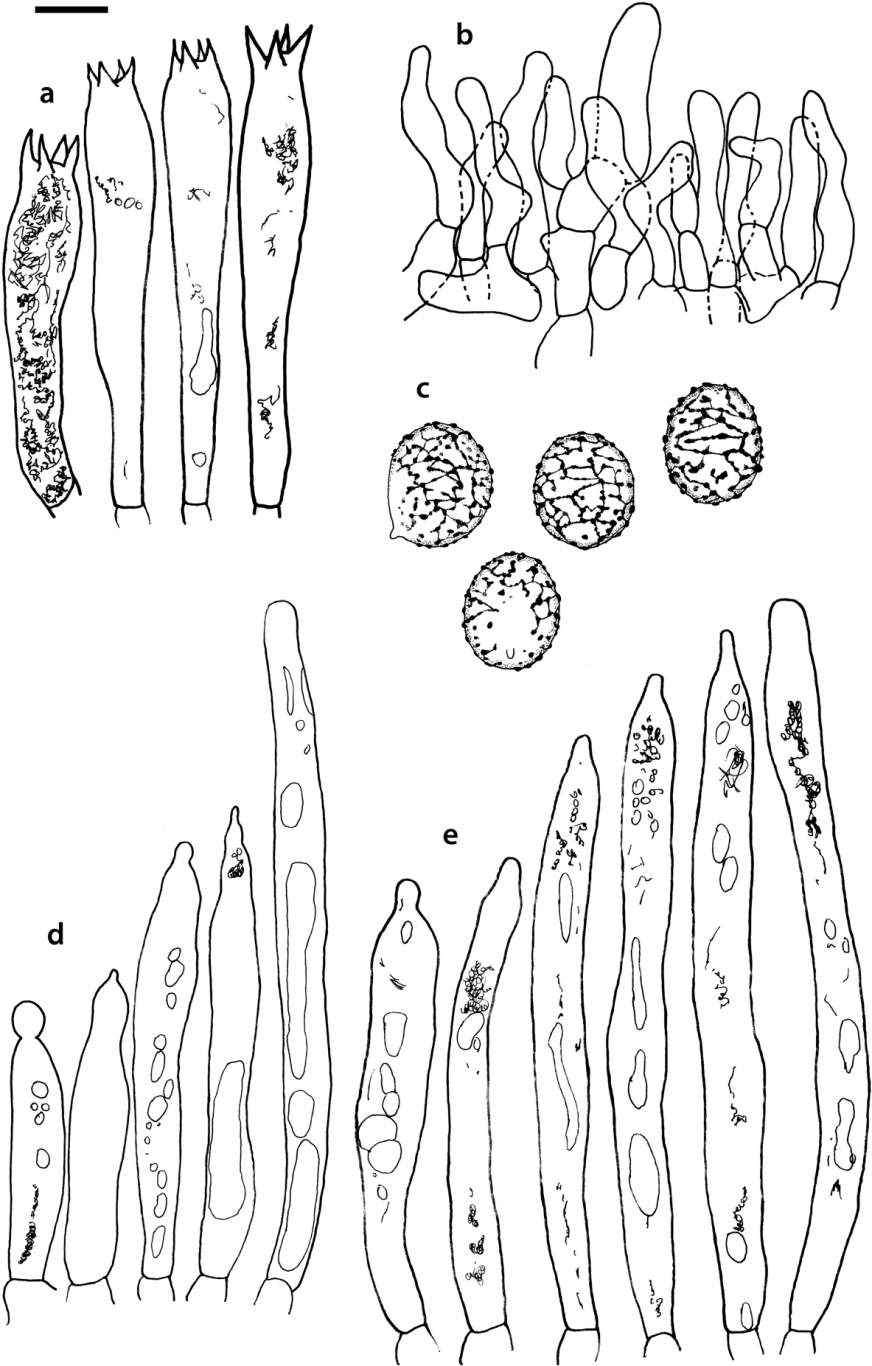
*Russula albonigra* (SAV F-755, SAV F-2559, SAV F-20177), hymenium. **a** Basidia. **b** Marginal cells. **c** Basidiospores. **d** Cystidia near lamellae edges. **e** Cystidia on lamellae sides. Bar = 10 μm, except for c 5 μm

**Fig. 5:**
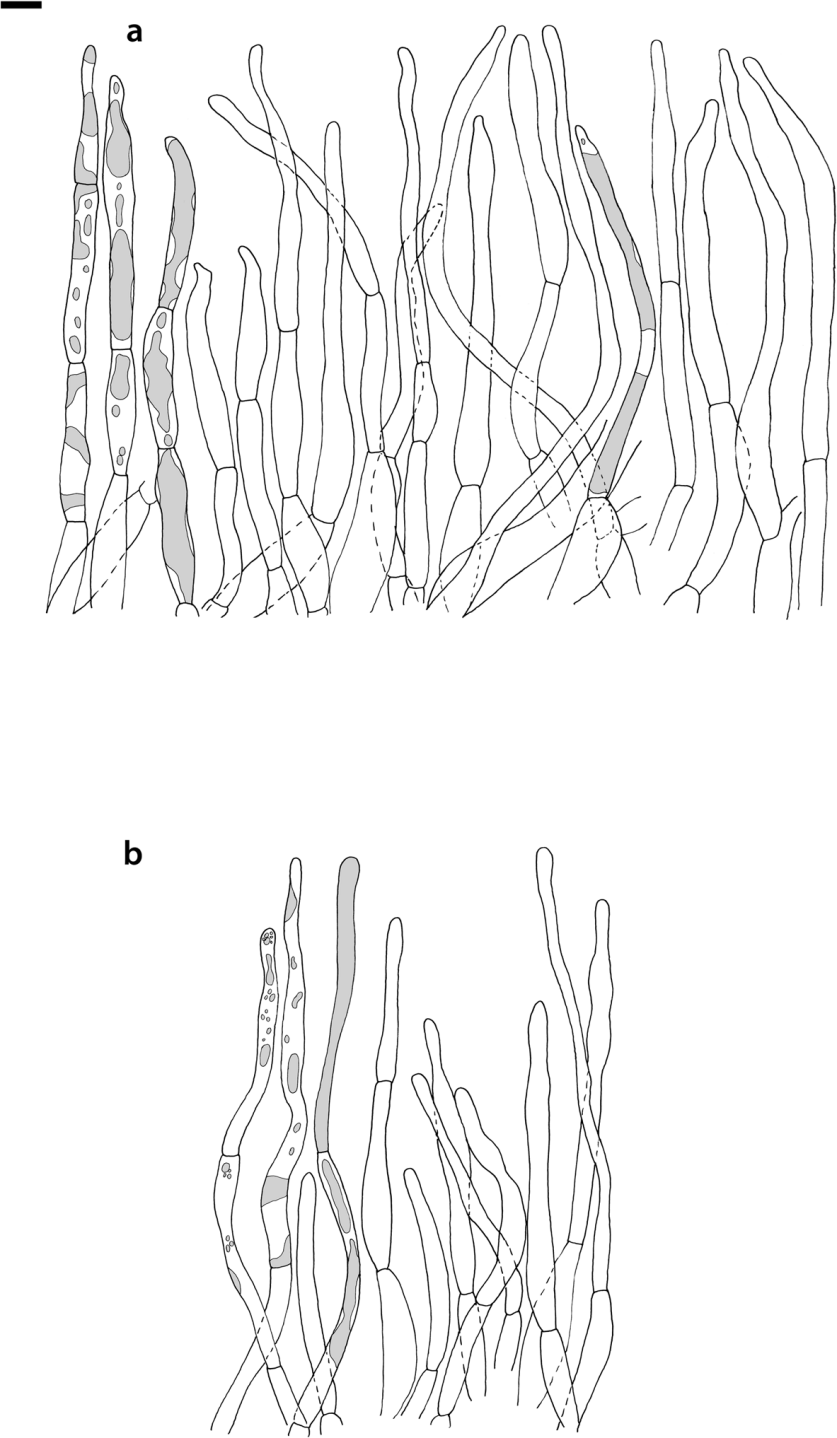
*Russula albonigra* (SAF F-755, SAV F-20177), hyphal terminations of the pileipellis. **a** Near the pileus margin. **b** Near the pileus centre. Bar = 10 μm

**Fig. 6:**
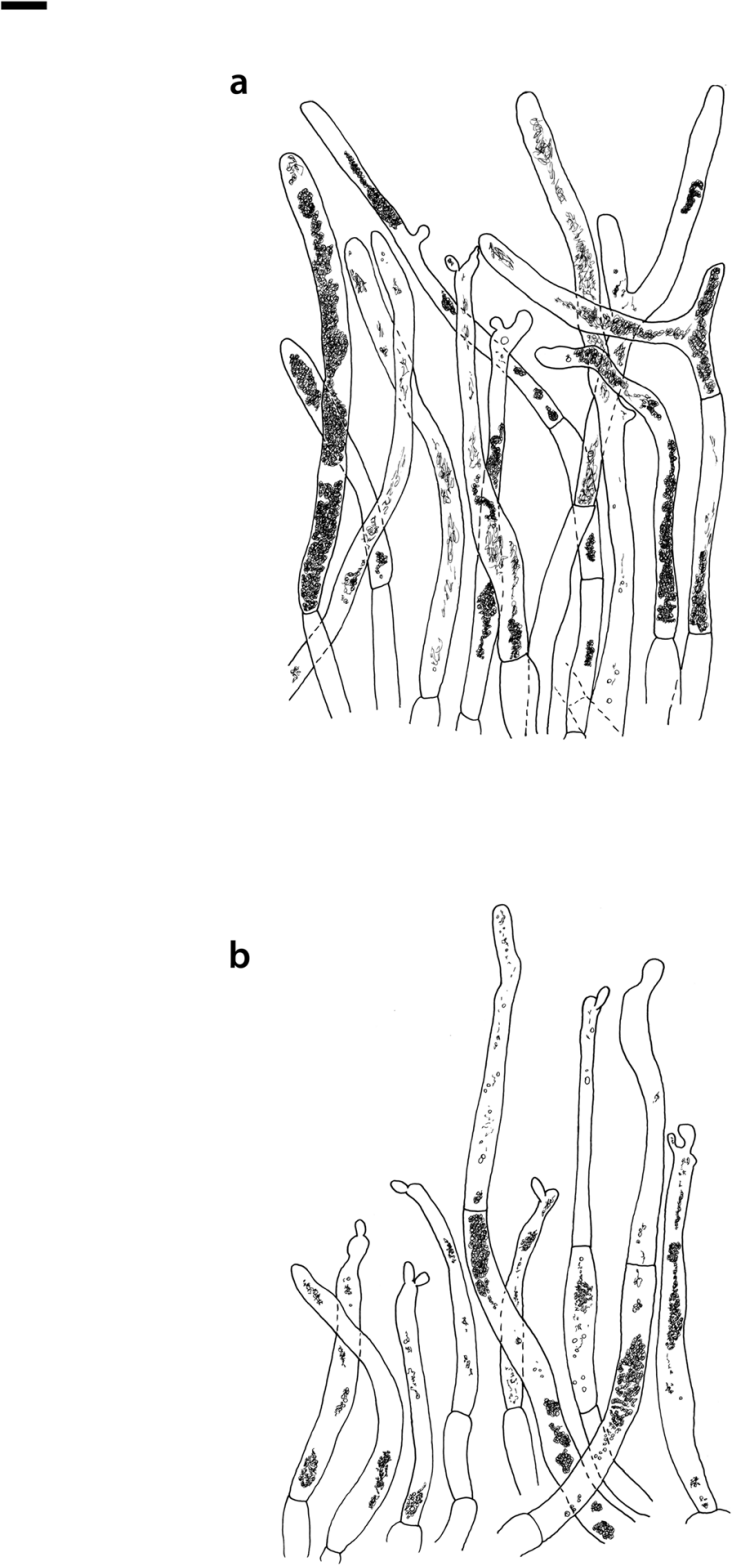
*Russula albonigra* (SAV F-20177), pileocystidia. **a** Near the pileus margin. **b** Near the pileus centre. Bar = 10 μm

***Russula albonigra*** (Krombh.) Fr., *Hymenomyc*. *eur*. (Upsaliae): 440 (1874). (Figs. 3c-f, 4, 5 and 6).

*Basionym*: *Agaricus alboniger* Krombh., *Naturgetr. Abbild. Beschr. Schwämme* (Prague) **9**: 27 (1845).

*Type*: Krombholz (1845) **lectotype designated here**, MB10000343); **Slovakia**: Oblík Nature reserve, with *Fagus*, 23 Sept. 2017, *S. Adamčík* (SAV F-20179 – **epitype designated here**, MB10000342).

*Description***:** *Pileus* large, 56–112 mm diam., plano-convex, at the centre with shallow but wide depression; margin deflexed, long involuted, not striated, smooth; pileus surface velvety and smooth near margin, towards the centre radially wrinkled or rugulose, centre smooth, dry, matt, almost not peeling (max. to 1/3 of the radius); young completely white, later becoming yellowish white (4A2), cream (4A3) to orange-grey (5B2) at the centre, more greying and blackening when old. *Lamellae* segmentiform to subventricose, to 6 mm deep, adnate to subdecurrent; snow white, later yellowish white (4A2), blackening with age or when bruised; with numerous lamellulae of different lengths, frequently forked near the stipe but also near the pileus margin, often anastomosed; moderately crowded to moderately distant, L = 190–260, l = 1 (one between each pair of long lamellae); edges even, concolorous, blackening with age. *Stipe* 42–60 × 14–29 mm, cylindrical, firm and fleshy, longitudinally striated, velvety near the lamellae; white, later becoming greyish orange (5B4) near the base; interior solid, cortex ca. 2.5 mm thick. *Context* ca. 7 mm thick at mid-radius, hard, white, turns rapidly grey and then black on cut section, at surface also turns red before grey and black; turning orange with FeSO_4_, immediately dark blue with guaiac (strong reaction, +++); taste mild, slightly like mint (refreshing) in lamellae, odour weak of apples. Spore print white (Ia).

*Basidiospores* (7.1–)7.5–*8.0*–8.5(− 9.4) × (5.6–)5.9–*6.3*–6.7(− 7.1) μm, broadly ellipsoid to ellipsoid, Q = (1.16–)1.22–*1.29*–1.36(− 1.49); ornamentation of low, dense [(6–)7–10(− 11) in a 3 μm diam circle] amyloid warts, 0.1–0.4 μm high, subreticulate, abundantly fused into chains [(0–)3–7(− 8) fusions in a 3 μm diam circle], and also connected by short, fine line connections [0–4(− 6) in a 3 μm diam circle]; suprahilar spot medium-sized, not amyloid. *Basidia* (44–)48.9–*55.1*–61.3(− 75) × 10.0–*10.8*–11.6(− 13) μm, narrowly clavate, 4-spored. *Hymenial cystidia* (65–)69.6–*85.8*–102.0(− 115) × (7–)7.6–*8.5*–9.4(− 10) μm, cylindrical to narrowly fusiform, apically obtuse to mucronate, thin-walled; with little content composed of large pale, oily, refringent guttules, without reaction in sulfovanillin; near the lamellae edges, (30–)40.4–*58.5*–76.6(− 98) × (6–)7.1–*7.9*–8.7(− 9) μm, cylindrical to narrowly fusiform, sometimes slightly flexuose, apically obtuse to mucronate or with small appendage, thin-walled, content as on lamellae sides. *Lamellae edges* sterile, when older elements can contain brown pigments; *marginal cells* (11–)16.5–*22.1*–27.7(− 31) × (3–)3.5–*4.9*–6.3(− 8) μm, poorly differentiated, cylindrical, flexuose, thin-walled. *Pileipellis* orthochromatic in Cresyl Blue, 80–90 μm deep, not sharply delimited from trama and not gradually passing, intermediate; subpellis not delimited from suprapellis; hyphae 3–6 μm wide near trama, not regular in width, dense, homogeneous, pigmented only near the surface, with no distinct gelatinous coating or only weakly on deeper hyphae. *Acid-resistant incrustations* absent. *Hyphal terminations* near the pileus margin long, with multiple septa, flexuous, thin-walled, filled with irregular refractive bodies containing brown pigments; terminal cells very long (35–)55.9–*86.6*–117.3(− 160) × (5–)5.2–*6.6*–8.0(− 10) μm, narrowly cylindrical to subulate, on average apically constricted to 3.5 μm (average difference of 3.2 μm between maximum width and width of the tips); subterminal cells and the cells below usually shorter and gradually wider, subterminal cells occasionally branched. Hyphal terminations near the pileus centre slightly slender and apically less attenuated; terminal cells slightly shorter (40–)51.8–*72.9*–94.0(− 124) × (4–)4.5–*6.0*–7.5(− 10) μm, subterminal cells never branched. *Pileocystidia* near the pileus margin widely dispersed, 1–3 celled, long, terminal cells (61–)73.0–*97.5*–122.0(− 160) × (5–)5.5–*7.0*–8.5(− 10) μm, cylindrical to subulate, flexuose, sometimes with small lateral projection, apically obtuse or with 1–2 eccentric appendages, sometimes bifurcating, with oily guttulate content, without reaction in sulfovanillin; near the pileus centre widely dispersed, 1–2 celled, generally shorter, terminal cells (52–)58.0–*75.2*–92.4(− 115) × (4–)5.1–*6.8*–8.5(− 11) μm, similar in shape and content, mostly apically with 1–2 eccentric appendages, not bifurcating. Pileocystidia not near surface but only deeper in the pileipellis. *Oleiferous hyphae* containing brown pigments and *cystidioid hyphae* present in the trama.

*Ecology*: Growing with *Fagus sylvatica*, *Abies alba, Picea abies,* and *Carpinus betulus*.

*Distribution*: Known from Austria, the Czech Republic, Estonia, Germany, Norway, and Slovakia.

*Additional material studied*: **Slovakia**: Stužica Natural reserve, under Kýčera hill, with *Abies* and *Fagus*, 22 Sept. 2017, *S. Adamčík*, (SAV F-20177); Stužica National Nature Reserve, central part, with *Fagus sylvatica*, 5 Oct. 2003, *S. Adamčík*, (SAV F-755); Badínsky prales Nature reserve, with *Abies* and *Fagus*, 22 Sept. 2017, *S. Adamčík*, (SAV F-2559); Badínsky prales Nature reserve, with *Abies* and *Fagus*, 29 Sept. 2011, *S. Adamčík*, (SAV F-3465); Revúca, road to Sirk, with *Fagus sylvatica*, 14 Oct. 2015, *S. Adamčík*, (SAV F-4776). **– Germany**: Bavaria, Oberallgäu, Oberstaufen, Hündle, alt. 975 m, N47°32′45.9″ E10°04′15.1″, with *Abies alba*, *Fagus sylvatica* and *Picea abies*, 6 Sept. 2013, *J. Kleine*, JK RUS 13090603 (hb. Jesko Kleine). **– Czech Republic**: Central Bohemia, Chrudim District, Bojanov (Horní Bezděkov), under *Fagus*, 12 Aug. 2012, *J. Borovička*, (PRM 934322); Central Bohemia, Kladno District, Běleč (Jenčov), under *Fagus* and *Carpinus*, 3 Sept. 2013, *J. Borovička*, (PRM 924409).

**Fig. 7:**
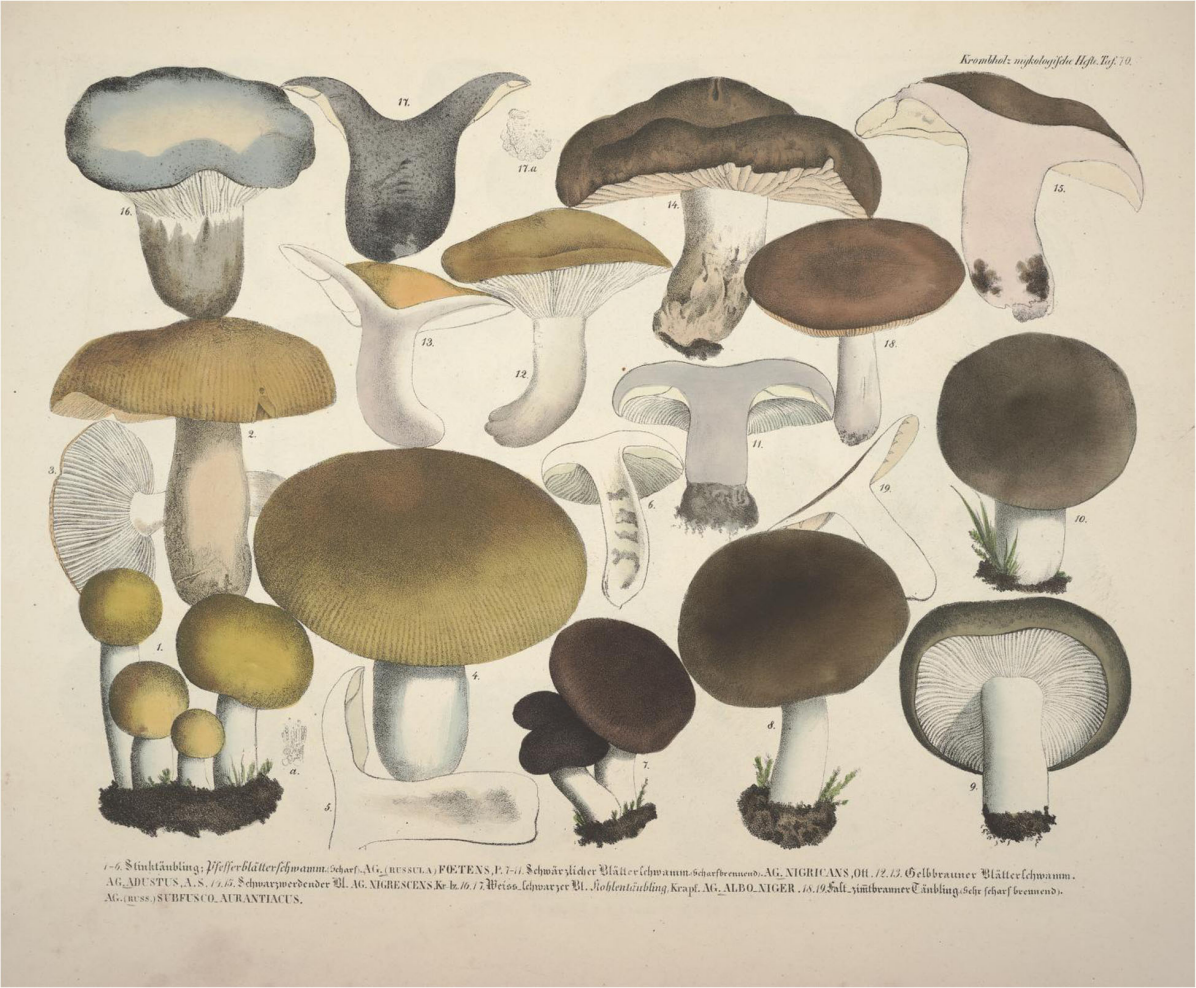
Krombholz (1845). Image from the BHL (Biodiversity Heritage Library). Contributed by Missouri Botanical Garden, Peter H. Raven Library

*Notes*: *Russula albonigra* was first described by Krombholz (1845) as *Agaricus alboniger* with only a brief description and no holotype designated. Later, Fries classified it in the genus *Russula* under its current name (Fries 1874) that became well-known and widely used in Europe. The illustration (Krombholz 1845: pl. 70, Figs. 16 and 17; Biodiversity Heritage Library), reproduced in Fig. 7 and made by Krombholz was suggested to serve as lectotype by Sarnari, although it was never formally designated (Sarnari 1998). The illustration is the only available original material and is hereby formally designated as the lectotype. The brief description and the plate itself are not sufficient to determine which species of the *R. albonigra* complex corresponds to *R*. *albonigra*. This makes the collecting area of Krombholz the most relevant criterion and in Krombholz (1845) it is mentioned that *R*. *albonigra* is found in Prague. Within the dataset, collection PRM 924409 was found only 31 km from the Prague city centre. Therefore, the clade in which this collection is placed, is chosen to represent *R*. *albonigra*. Specimen SAV F-20197 is here designated as epitype.

**Fig. 8:**
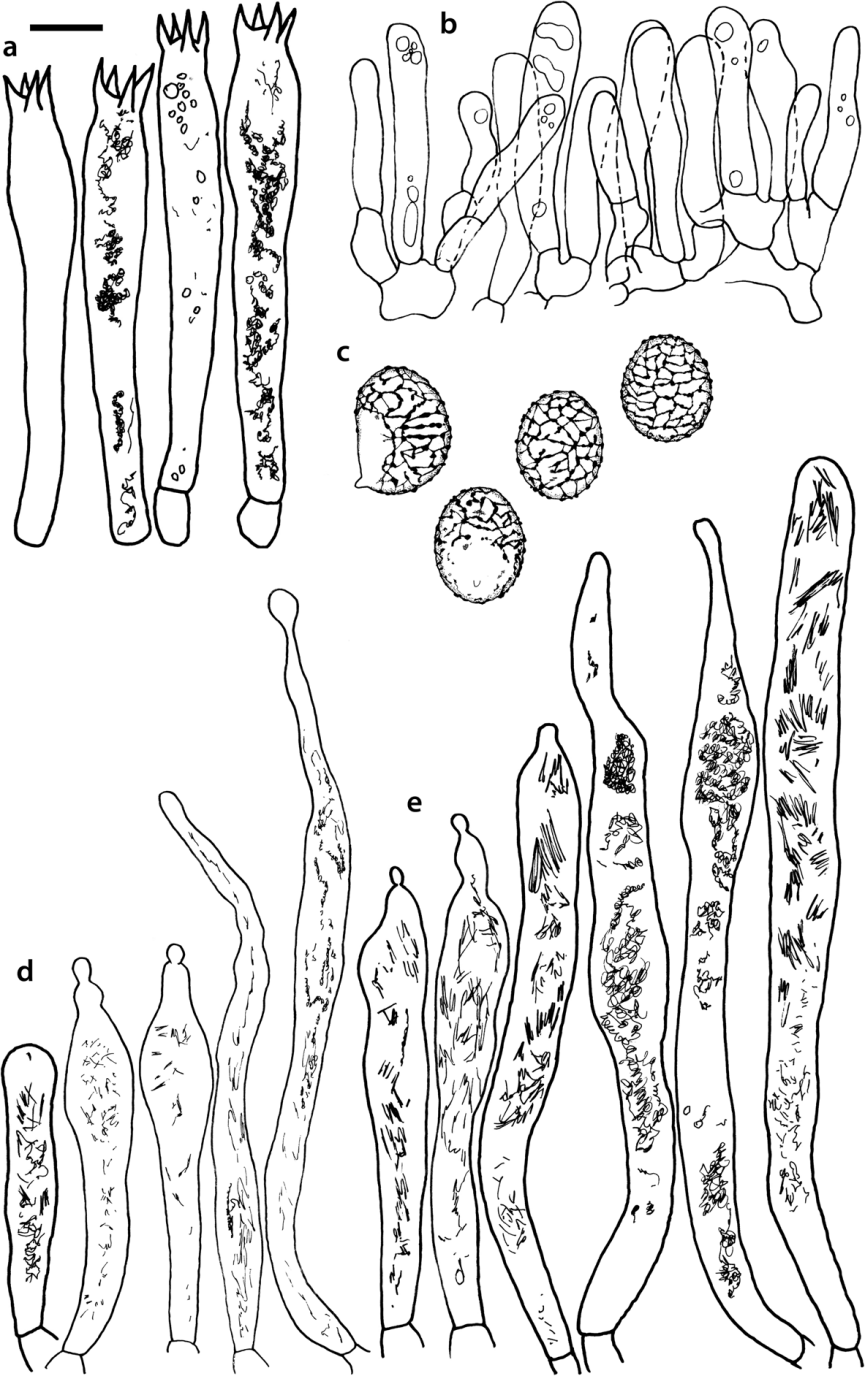
*Russula ambusta* (SAV F-3358, FH 2008 ST01), hymenium. **a** Basidia. **b** Marginal cells. **c** Basidiospores. **d** Cystidia near lamellae edges. **e** Cystidia on lamellae sides. Bar = 10 μm, except for c 5 μm

**Fig. 9:**
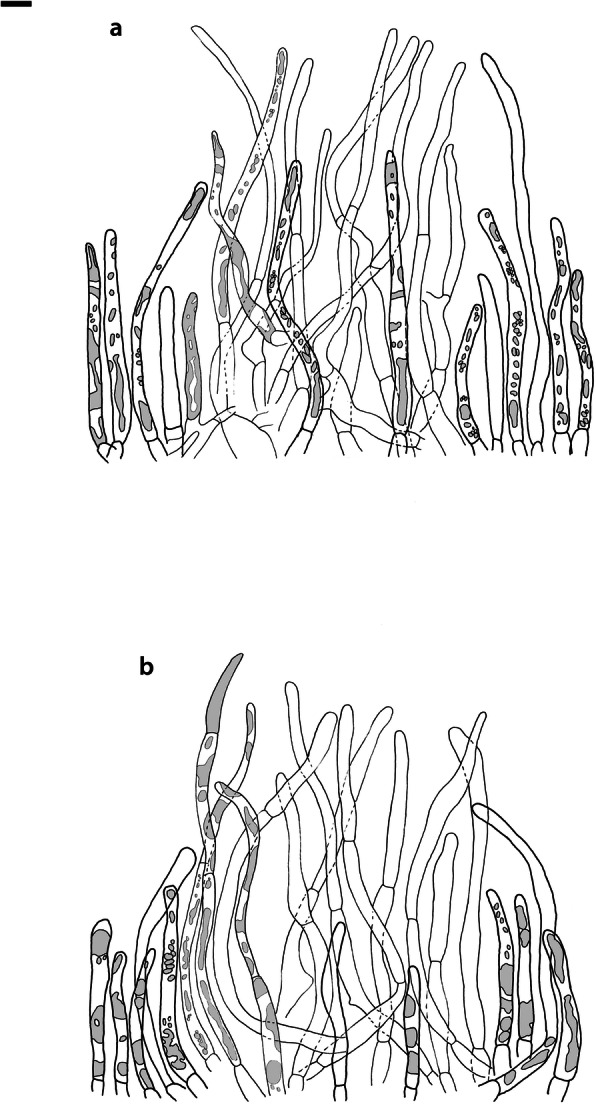
*Russula ambusta* (SAV F-3358, FH 2008 ST01), hyphal terminations of the pileipellis. **a** Near the pileus margin. **b** Near the pileus centre. Bar = 10 μm

**Fig. 10:**
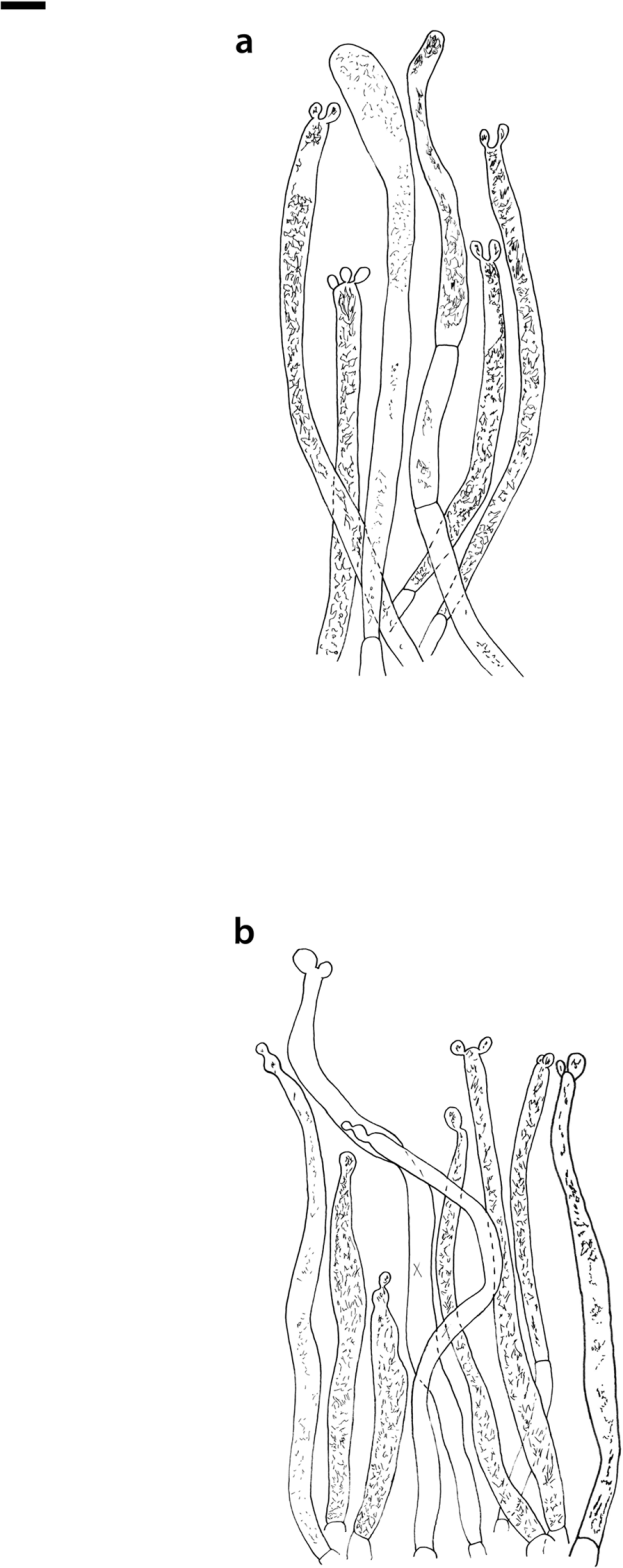
*Russula ambusta* (SAV F-3358), pileocystidia. **a** Near the pileus margin. **b** Near the pileus centre. Bar = 10 μm

***Russula ambusta*** De Lange, Adamčík & F. Hampe, **sp. nov.** (Figs. 3g, 8, 9 and 10).

MycoBank: MB839080.

*Etymology*: Refers to the appearance of the basidiomata, which look like they were burnt.

*Diagnosis*: Differs from the other species of the *Russula albonigra* complex by the intermediate reticulation and density of spore ornamentation and the presence of appendages, but lack of bifurcations on the pileocystidia.

*Type*: **Slovakia**: Vývrať, Bučková, W slopes of the hill, with *Quercus*, 6 July 2011, *V. Kučera* (SAV F-3558 – holotype).

*Description: Pileus* large, 45–100 mm diam, planoconvex to applanate, centrally depressed to umbilicate, becoming more infundibuliform when older; margin slightly inflexed when young, straight when mature, smooth; pileus surface smooth, dry, dull to somewhat viscid when wet; greyish orange, light brown (5B5, 5D5) to umber, greyish brown (5F8, 6F3) with paler white to sand coloured patches. *Lamellae* segmentiform to subventricose, to 6 mm deep, adnate to subdecurrent; snow white, later yellowish white (4A2), blackening with age or when bruised; with numerous lamellulae of different lengths; dense (7–10 L + 5–8 l/cm at mid-radius); edges even, concolorous, blackening with age. *Stipe* 30–40 × 15–25 mm, cylindrical, firm and fleshy, smooth; white but rapidly almost completely orange brown; solid inside. *Context* ca. 3–5 mm thick at mid-radius, firm, white, greying before blackening, no reddening observed; turning immediately dark blue with guaiac (strong reaction, +++); taste first mild, then quickly somewhat cooling with menthol component, never spicy or acrid; odour indistinct. *Spore print* white (Ia).

*Basidiospores* (6.7–)7.4–*8.2*–9.0(− 9.6) × (5.4–)5.7–*6.0*–6.3(− 6.8) μm, broadly ellipsoid to narrowly ellipsoid, Q = (1.18–)1.28–*1.38*–1.48(− 1.54); ornamentation of very low, dense to very dense [(6–)7–13(− 16) in a 3 μm diam circle] amyloid warts, up to 0.2 μm high, subreticulate to reticulate, abundantly fused in chains [(2–)3–8(− 10) fusions in a 3 μm diam circle], and also connected by short, fine line connections [(0–)1–9(− 17) in a 3 μm diam circle]; suprahilar spot medium-sized, not amyloid. *Basidia* (54–)57.5–*62.6*–67.7(− 76) × (8–)8.9–*9.5*–10.1(− 11) μm, narrowly clavate, 4-spored. *Hymenial cystidia* (66–)67.1–*91.9*–107.7(− 125) × (7–)8.5–*9.8*–11.1(− 13) μm, variable: (1) narrowly fusiform to narrowly clavate, flexuose to even slightly moniliform, apically obtuse or with a constriction or with small appendage to even slightly mucronate, thin-walled; with heteromorphous, oily content, mostly fragmented in multiple crystalline-like masses or slightly granulose, without reaction in sulfovanillin; (2) narrowly fusiform to lanceolate, flexuose, tapering towards the top; with less content, heteromorphous, oily, mostly fragmented in multiple crystalline-like masses or slightly granulose, without reaction in sulfovanillin; near the lamellae edges, (29–)37.0–*63.7*–90.4(− 144) × (5–)7.2–*8.5*–9.8(− 10) μm, (1) narrowly fusiform to narrowly clavate, flexuose, apically obtuse or tapering towards the top in a moniliform way with a small appendage, thin-walled; content as on lamellae sides; (2) as on lamellae sides. *Lamellae edges* sterile, when older elements can contain brown pigments; *marginal cells* (15–)19.1–*26.1*–33.1(− 36) × (4–)4.7–*5.6*–6.5(− 7) μm, undifferentiated, cylindrical to narrowly clavate, thin-walled. *Pileipellis* orthochromatic in Cresyl Blue, 175–400 μm deep, not sharply delimited from trama, gradually passing; subpellis not delimited from suprapellis; hyphae 3–6 μm wide near trama, dense near surface and near trama, irregularly oriented, more parallel and horizontal near trama and surface, intricate everywhere, pigmented near surface only, with no distinct gelatinous coating. *Acid-resistant incrustations* absent. *Hyphal terminations* near the pileus margin long, with multiple septa, flexuous, thin-walled, filled with irregular refractive bodies containing brown pigments; terminal cells (41–)51.4–*70.2*–89.0(− 125) × (3–)4.3–*5.4*–6.5(− 8) μm, narrowly cylindrical, on average apically constricted to 3.5 μm; subterminal cells and the cells below similar in length or slightly shorter, similar in width or gradually slightly wider, subterminal cells and cells below sometimes branched. Hyphal terminations near the pileus centre similar, terminal cells usually shorter (28–)40.8–*59.8*–78.8(− 106) × (3–)4.0–*5.0*–6.0(− 7) μm, subterminal cells and cells below not branching. *Pileocystidia* hard to find; near the pileus margin widely dispersed to rare, mostly 1-celled, but up to 3-celled, long, terminal cells (73–)79.1–*110.6*–142.1(− 145) × 4.9–*7.2*–9.5(− 11) μm, cylindrical, flexuose, apically mostly with 2–3 eccentric appendages or obtuse, content as in hymenial cystidia or more granulose, without reaction in sulfovanillin; near the pileus centre widely dispersed, 1-celled, (63–)79.4–*106.7*–134.0(− 151) × (4–)5.0–*6.4*–7.8(− 8) μm, cylindrical to slightly subulate, apically with double constriction or 1–2 eccentrical appendages, content as near pileus margin. *Oleiferous hyphae* containing brown pigments and *cystidioid hyphae* present in the trama.

*Ecology*: Growing with *Quercus robur, Pinus sylvestris,* and *Betula pendula*.

*Distribution*: Known from Estonia, Germany, Slovakia, Spain, and Sweden.

*Additional material studied*: **Germany**: Brandenburg, Landkreis Oder-Spree, near Helenesee Frankfurt-Oder, Markendorfer Forst, MTB 3752/2 (Müllrose), N 52.256386 E 14.46049, lichen-pine (*Pinus sylvestris*) forest with interspersed birch trees (*Betula pendula*) on an unpaved forest path, on sandy soil, 19 Oct. 2008, *F. Hampe* & *I. Kindermann*, FH 2008 ST01 (hb. Felix Hampe).

*Notes*: The range of the spore size is large within this species. This results from the spore size difference between the collections. The holotype (SAV F-3558) has smaller spores and lower Q-value than collection FH 2008 ST01. The variability in the shape of the hymenial cystidia is also due to the difference between the collections with the holotype having cystidia of type 1 and collection FH 2008 ST01 having cystidia of type 2 (types referring to (1) and (2) in the description). Therefore, based on morphology, we could hypothesize that these collections represent different species. Nevertheless, we treat them here as the same species because phylogenetically there is no support for the hypothesis of two different species. All markers used in this study place these two collections together as the same species. Measurements are given for each collection separately in Supplementary Material 1. Of course, more collections and more micromorphological study are needed in order to understand this intraspecific variation. The UNITE species hypothesis at 0.5% corresponding to our concept of *R. ambusta* is based on a locked sequence and after this publication we will propose to change the reference sequence to the holotype of the species.

**Fig. 11:**
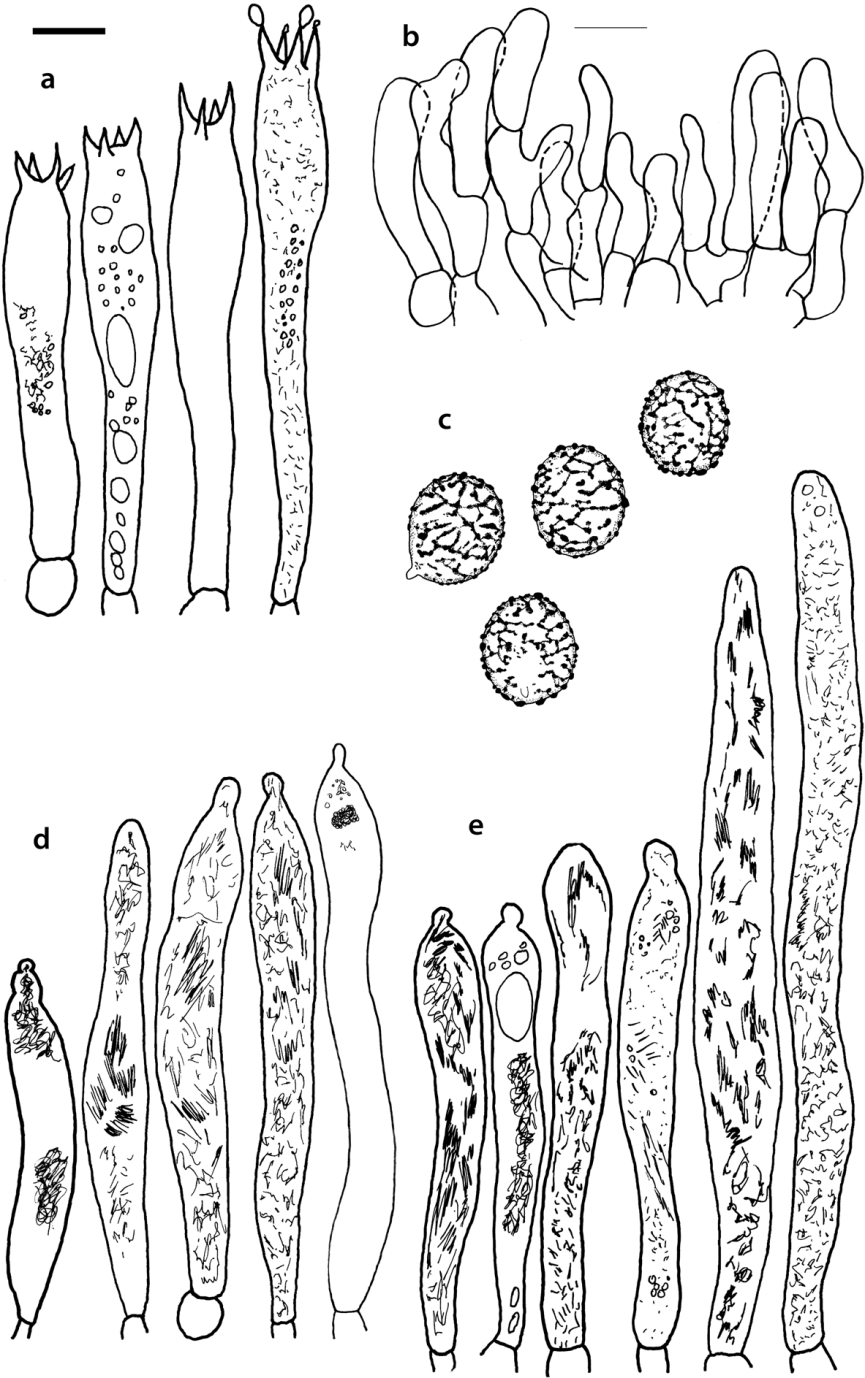
*Russula nigrifacta* (RDL 16–028, RDL 16–044/2, RDL 16–063, SAV F-2418), hymenium. **a** Basidia. **b** Marginal cells. **c** Basidiospores. **d** Cystidia near lamellae edges. **e** Cystidia on lamellae sides. Bar = 10 μm, except for c 5 μm

**Fig. 12:**
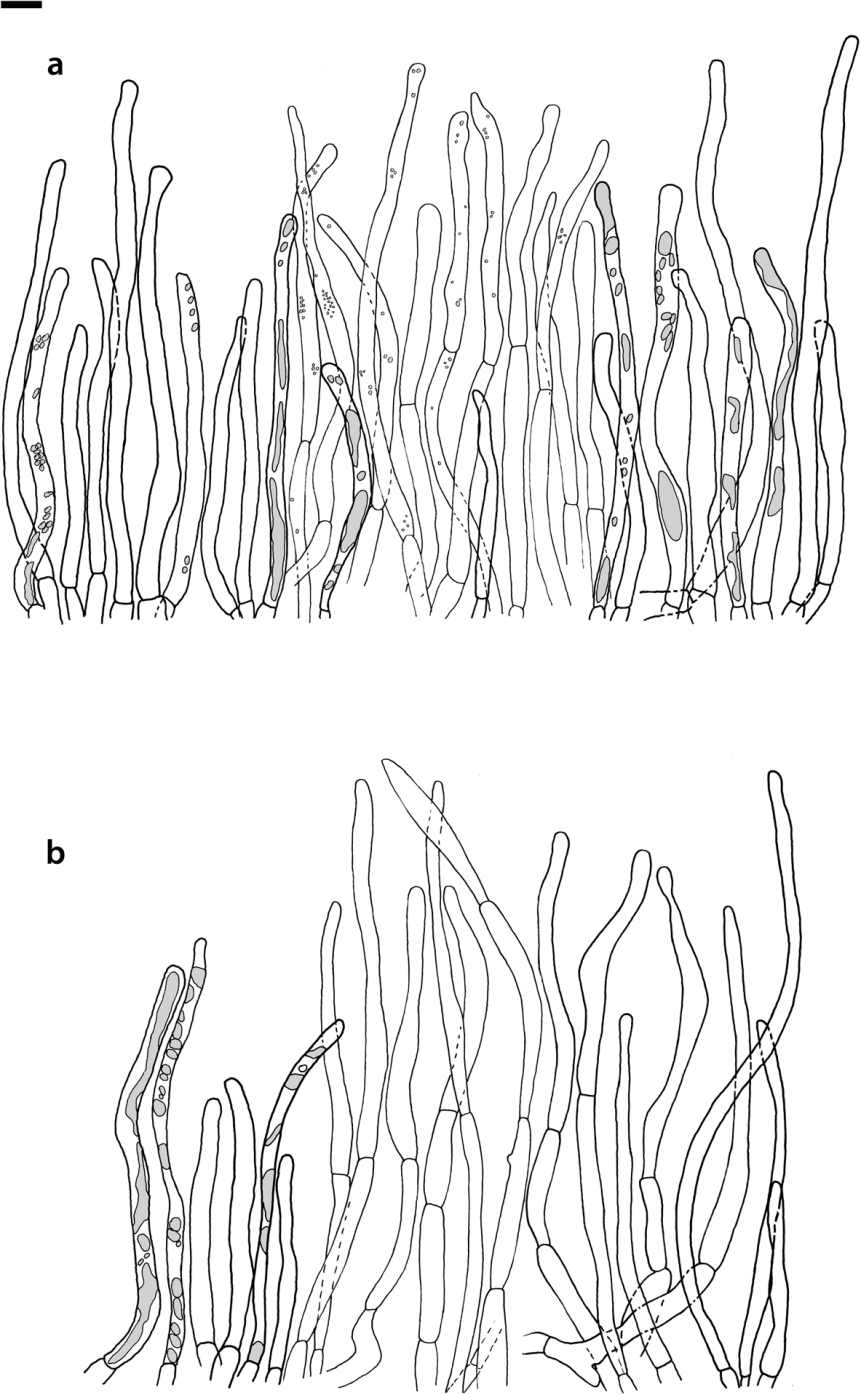
*Russula nigrifacta* (RDL 16–028, RDL 16–044/2, RDL 16–063, SAV F-2418, SAV F-2419), hyphal terminations of the pileipellis. **a** Near the pileus margin. **b** Near the pileus centre. Bar = 10 μm

**Fig. 13:**
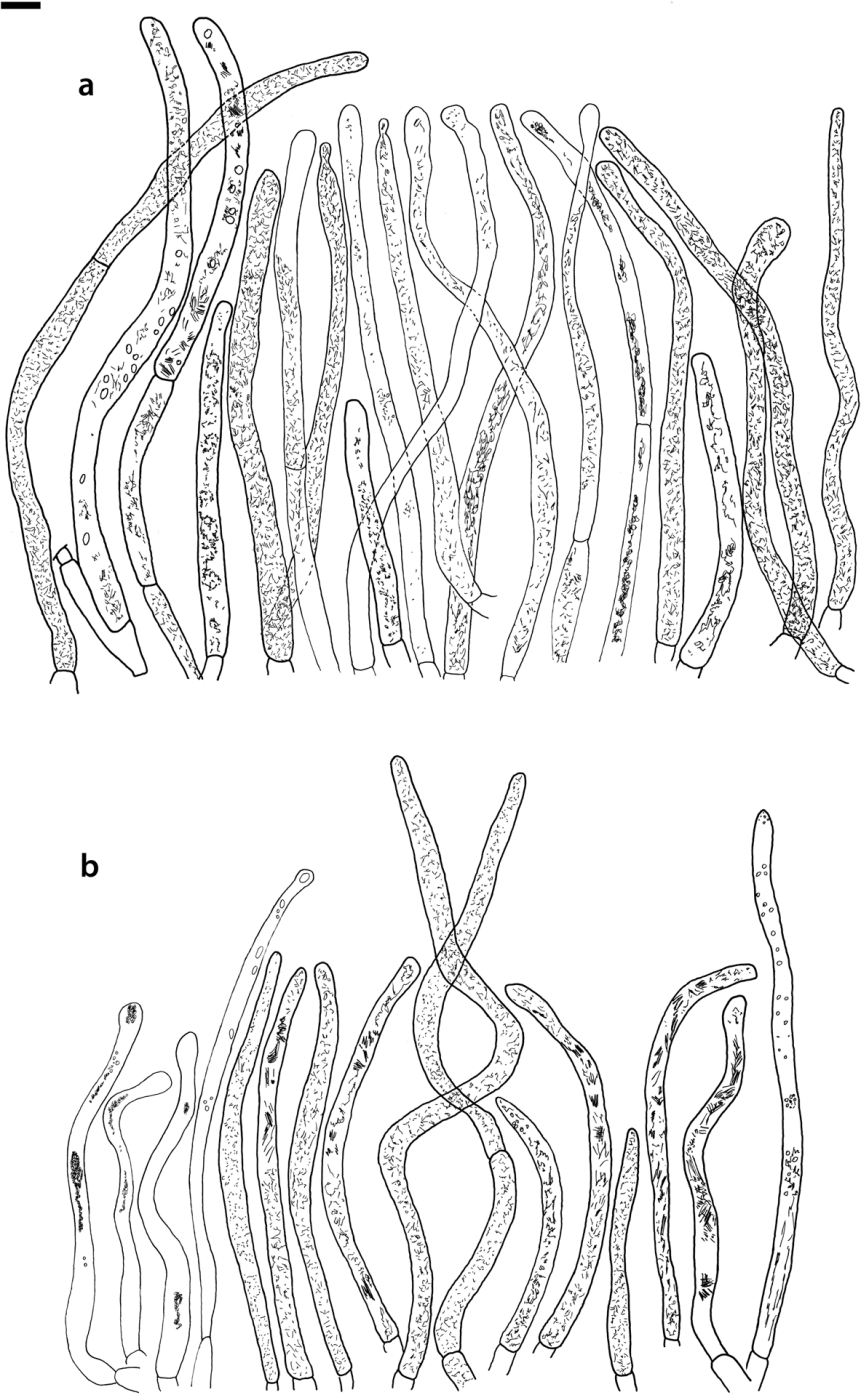
*Russula nigrifacta* (RDL 16–028, RDL 16–044/2, RDL 16–063, SAV F-2418, SAV- F-2419), pileocystidia. **a** Near the pileus margin. **b** Near the pileus centre. Bar = 10 μm

***Russula nigrifacta*** De Lange & Adamčík, **sp. nov.** (Figs. 3a-b, 11, 12 and 13).

MycoBank: MB839081.

*Etymology*: Named after the strong blackening of the basidiomata.

*Diagnosis*: Differs from the other species of the *Russula albonigra* complex by the lack of both appendages and bifurcations on the pileocystidia.

*Type*: **Italy**: Tuscany, Province of Livorno, Piombino, with *Quercus ilex* and *Quercus suber*, 9 Nov. 2016, *R. De Lange*, RDL 16–044 (GENT – holotype).

*Description****:*** *Pileus* large, 75–105 mm diam, planoconvex to applanate, centrally depressed to umbilicate, becoming more infundibuliform when older; margin slightly inflexed when young, straight when mature, smooth; pileus surface smooth, sometimes slightly cracked at the margin, dry, dull to somewhat viscid when wet; ivory to cream, sand coloured, yellowish white (4A2, 5A3, 5B3) with darker spots of pale brownish/greyish orange, yellowish brown to light brown, dark brown (5B6, 5E4, 5E8, 5F4). *Lamellae* narrow, segmentiform to subventricose, 2–4 mm deep, adnate to subdecurrent; white to yellowish white, rarely with faintly blueish shine, blackening with age; with numerous lamellulae of different lengths without clear regular pattern, rarely locally anastomosing; dense (6–9 L + 8–9 l/cm at mid-radius); edges even, concolorous, blackening with age. *Stipe* 30–60 × 19–30 mm, cylindrical, firm and fleshy, smooth; white, becoming more orange brown with age; solid inside. *Context* ca. 6–7 mm thick at mid-radius, firm, white, blackening without reddening, surface of pileus and stipe also slightly reddening before blackening; turning greenish with FeSO_4_, yellowish with KOH, immediately dark blue with guaiac (strong reaction, +++); taste mild to slightly refreshing; odour fruity, sweet. *Spore print* white (Ia).

*Basidiospores* (6.7–)7.5–*8.0*–8.5(− 9.5) × (5.0–)5.7–*6.0*–6.3(− 7.0) μm, broadly ellipsoid to ellipsoid, Q = (1.15–)1.25–*1.34*–1.43(− 1.56); ornamentation of low, dense [(5–)7–10(− 11) in a 3 μm diam circle] amyloid warts, 0.2–0.4 μm high, subreticulate, abundantly fused into chains [(0–)3–6(− 8) fusions in a 3 μm diam circle] and also connected by short, fine line connections [0–3(− 4) in a 3 μm diam circle]; suprahilar spot small, not amyloid. *Basidia* (50–)57.0–*62.8*–68.5(− 79) × (8–)8.7–*9.6*–10.5(− 11) μm, narrowly clavate, 4-spored. *Hymenial cystidia* (57–)62.6–*82.6*–102.6(− 128) × (7–)7.7–*8.9*–10.1(− 15) μm, cylindrical to narrowly fusiform to narrowly clavate, sometimes slightly flexuose, apically obtuse or with small appendage, thin-walled; with heteromorphous, oily content, fragmented in multiple crystalline-like masses, without clear reaction in sulfovanillin; near the lamellae edges, (37–)53.0–*69.4*–85.8(− 119) × (7–)8.0–*9.1*–10.2(− 11) μm, cylindrical to narrowly fusiform, sometimes narrowly clavate, often slightly flexuose, apically obtuse with small appendage or mucronate to tapering towards the top, thin-walled; content as on lamellae sides. *Lamellae edges* sterile, when older elements can contain brown pigments; *marginal cells* (15–)17.7–*22.2*–26.7(− 30) × (4–)4.6–*5.9*–7.2(− 10) μm, poorly differentiated, cylindrical to narrowly clavate, flexuose, thin-walled. *Pileipellis* orthochromatic in Cresyl Blue, 90–136 μm deep, not sharply delimited from trama and not gradually passing, intermediate; subpellis not well delimited from suprapellis; hyphae 2.5–7 μm wide near trama, dense near surface and near trama, irregularly oriented, more parallel and horizontal near trama, pigmented throughout the pileipellis, with some gelatinous coating. *Acid-resistant incrustations* absent. *Hyphal terminations* near the pileus margin long, slender, with multiple septa, flexuous, thin-walled, filled with irregular refractive bodies containing brown pigments; terminal cells (50–)63.5–*83.5*–103.5(− 133) × (3–)4.0–*4.9*–5.8(− 7) μm, narrowly cylindrical; subterminal cells and the cells below similar in length and width, subterminal cells never branched. Hyphal terminations near the pileus centre similar; terminal cells (42–)53.1–*79.6*–106.1(− 135) × (3–)3.4–*4.6*–5.8(− 8) μm, sometimes more subulate, subterminal cells and cells below shorter, subterminal cells rarely branched. *Pileocystidia* near the pileus margin dispersed, 1–4 celled, very long, terminal cells (41–)83.4–*119.5*–155.6 (− 235) × (5–)5.8–*6.7*–7.6(− 9) μm, cylindrical, flexuose, apically obtuse or with slight constriction, with oily granulose content, without clear reaction in sulfovanillin; near the pileus centre dispersed, 1–2 celled, similar in shape and content or with less and more oily guttulate content, terminal cells (64–)80.9–*123.0*–165.1(− 220) × (4–)5.2–*6.0*–6.8(− 7) μm. *Oleiferous hyphae* containing brown pigments and *cystidioid hyphae* present in the trama.

*Ecology*: Growing with Mediterranean oaks (*Quercus ilex* and *Quercus suber*) in Italy, in Slovakia with *Quercus robur,* and *Carpinus betulus*.

*Distribution*: Known from Estonia, Italy, and Slovakia.

*Additional material studied*: **Italy**: Tuscany, Province of Livorno, Piombino, with *Quercus ilex* and *Quercus suber*, 7 Nov. 2016, *R. De Lange*, RDL 16–028 (GENT); Tuscany, Province of Livorno, Piombino, with *Quercus ilex* and *Quercus suber*, 11 Nov. 2016, *R. De Lange*, RDL 16–063 (GENT). **– Slovakia**: Obyce, forest NE of the village, with *Quercus* and *Carpinus*, 24 July 2008, *S. Adamčík*, (SAV F-2418); Prenčov, Horné Majere, with *Quercus* and *Carpinus*, 22 July 2008, *S. Adamčík*, (SAV F-2419); Tepličky, with *Quercus* and *Carpinus*, 4 July 2009, *S. Adamčík*, (SAV F-3006); Bohunický Roháč, forest close to the nature reserve, with *Quercus*, 8 Sept. 2006, *S. Adamčík*, (SAV F-1501).

*Notes*: Besides the diagnostic features mentioned in the diagnosis, *R. nigrifacta* is characterised by its higher spore ornamentation (incomplete reticulum) and the thin pileipellis. These features are shared with *R*. *albonigra*, which is easily differentiated from the other species in the complex by the oily guttulate content of the cystidia. Furthermore, our data suggests that *R*. *nigrifacta* is associated with *Quercus* spp. (and possibly also with *Carpinus betulus*) in thermophilous habitats (thermophilous oak forests and Mediterranean oak forests).

**Fig. 14:**
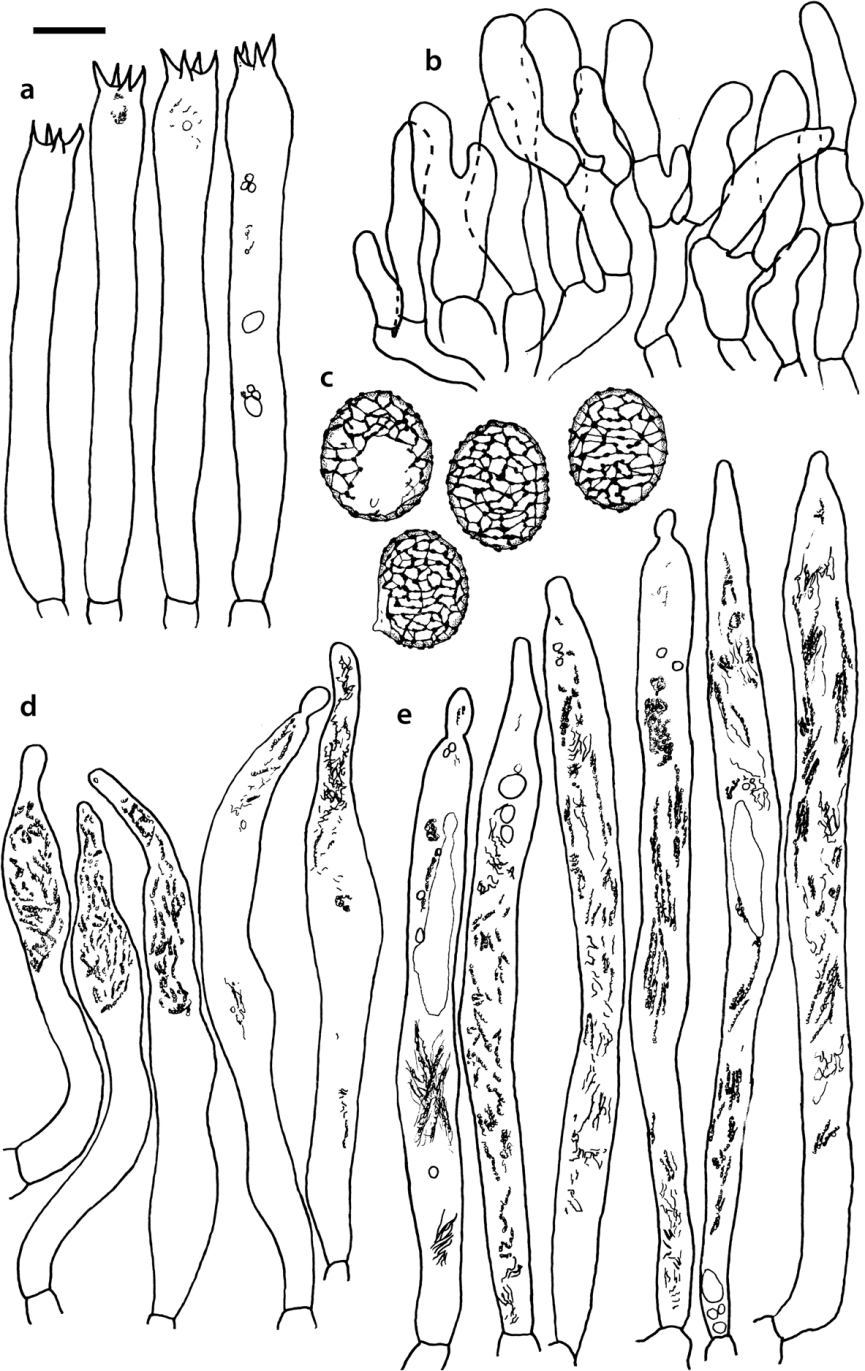
*Russula ustulata* (AV 16–019), hymenium. **a** Basidia. **b** Marginal cells. **c** Basidiospores. **d** Cystidia near lamellae edges. **e** Cystidia on lamellae sides. Bar = 10 μm, except for c 5 μm

**Fig. 15:**
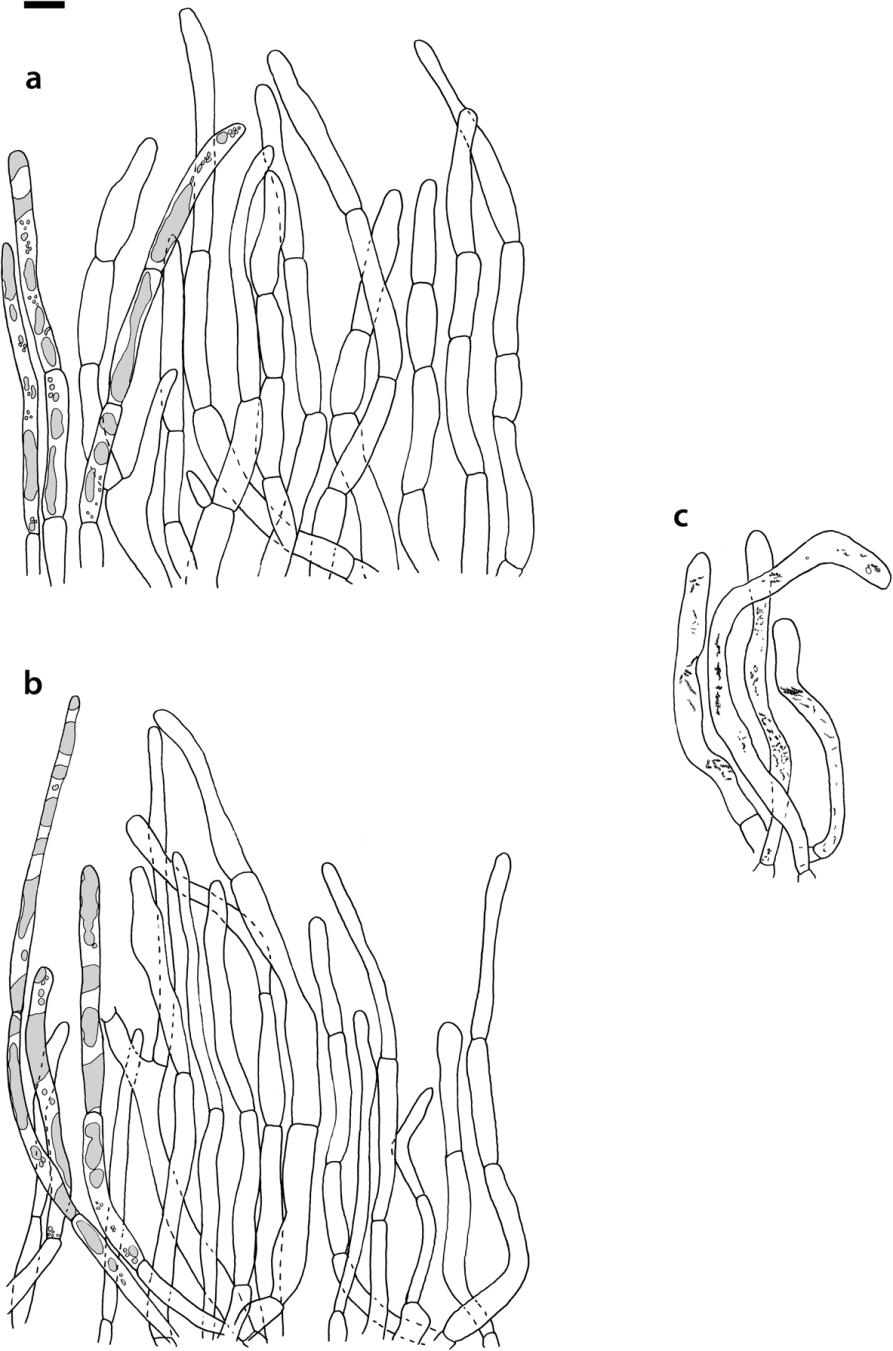
*Russula ustulata* (AV 16–019), pileipellis. **a** Hyphal terminations near the pileus margin. **b** Hyphal terminations near the pileus centre. **c** Pileocystidia near the pileus margin. Bar = 10 μm

***Russula ustulata*** De Lange & Verbeken, **sp. nov.** (Figs. 3h, 14 and 15).

MycoBank: MB839082.

*Etymology*: Refers to the appearance of the basidiomata, which look like they were burnt.

*Diagnosis*: Differs from the other species of the *Russula albonigra* complex by the absence (or rareness) of pileocystidia.

*Type*: **Norway**: NT Steinkjer, Kvamsfjellet, North of Lystjörna, Austerolsenget, alt. 137.5 m, N64°12′47″ E11°49′09″, 20 Aug. 2016, *A. Verbeken*, AV 16–019 (GENT – holotype).

*Description*: *Pileus* large, 101–112 mm diam, planoconvex to applanate, centrally depressed to umbilicate to infundibuliform, widely V-shaped; margin straight, smooth; pileus surface smooth, glabrous, dry, shiny; very dark blackish brown to dark grey-black (7F2–4) without lighter brown tints, uniform. *Lamellae* narrow, segmentiform to subventricose, 4–5 mm deep, adnate to subdecurrent; completely white to yellowish white, quickly blackening; with numerous lamellulae of different lengths, locally anastomosing; moderately distant (6–9 L + 3–4 l/cm at mid-radius) to distant (sometimes almost as in *R. nigricans*); edges even, papery thin, concolorous, quickly blackening with age. *Stipe* 45–65 × 25–30 mm, cylindrical, firm and fleshy, smooth to irregular surface, dry; white but rapidly turning dark grey, black (stays whitish only at the top); solid inside. *Context* ca. 5–9 mm thick at mid-radius, firm, white, staining grey, then brownish black, mostly blackening without reddening but a slight pink tinge can be present; turning greenish with FeSO_4_; taste mild but not agreeable, musty, but slightly menthol-like in the gills. *Spore print* white (Ia).

*Basidiospores* (7.4–)8.1–*8.5*–8.9(− 9.3) × (5.7–)6.0–*6.3*–6.6(− 6.9) μm, broadly ellipsoid to ellipsoid, Q = (1.12–)1.26–*1.36*–1.46(− 1.48); ornamentation of very low, very dense [(9–)10–15(− 17) in a 3 μm diam circle] amyloid warts, to 0.2 μm high, reticulate, abundantly fused into chains [3–7(− 10) fusions in a 3 μm diam circle], abundantly connected by short, fine line connections [(6–)7–11(− 13) in a 3 μm diam circle]; suprahilar spot large, not amyloid. *Basidia* (53–)58.9–*67.3*–75.7(− 83) × (8–)8.4–*9.2*–10.0(− 11) μm, narrowly clavate to cylindrical, 4-spored. *Hymenial cystidia* (78–)88.7–*106.6*–124.5(− 153) × (7–)8.0–*9.1*–10.2(− 12) μm, cylindrical to narrowly fusiform, apically obtuse with small appendage or slightly tapering towards the top, thin-walled; with heteromorphous, oily content, fragmented in multiple masses to needle-like crystalline, without reaction in sulfovanillin; near the lamellae edges, (60–)70.0–*82.9*–95.8(− 111) × (7–)8.1–*9.5*–10.9(− 14) μm, narrowly fusiform to narrowly conical, slightly flexuose, apically obtuse, sometimes with small appendage or tapering towards the top, thin-walled; content as on lamellae sides, often containing brown pigments. *Lamellae edges* sterile, when older elements can contain brown pigments; *marginal cells* (14–)19.8–*26.5*–33.2(− 39) × 4.9–*6.3*–7.7(− 10) μm, poorly differentiated, cylindrical to narrowly clavate to fusiform, flexuose, thin-walled. *Pileipellis* orthochromatic in Cresyl Blue, 250–300 μm deep, not sharply delimited, gradually passing; subpellis not delimited from suprapellis; hyphae 3–7 μm wide near trama, dense near surface and near trama, loose in intermediate zone, irregularly oriented, pigmented throughout the pileipellis, some gelatinous coating can be present deeper in the pileipellis. *Acid-resistant incrustations* absent. *Hyphal terminations* near the pileus margin long, with multiple septa, flexuous, thin-walled, filled with irregular refractive bodies containing brown pigments; terminal cells (29–)36.0–*53.6*–71.1(− 100) × (4–)4.9–*6.0*–7.1(− 9) μm, narrowly cylindrical to subulate, on average apically constricted to 4.5 μm; subterminal cells and the cells below shorter and gradually wider, subterminal cells never branched, cells below occasionally branched. Hyphal terminations near the pileus centre slightly slender and apically less attenuated; terminal cells slightly longer, (31–)43.3–*59.0*–74.7(− 89) × (3–)4.0–*5.1*–6.2(− 8) μm, subterminal cells and cells below more of similar size as terminal cells, subterminal cells rarely branched. *Pileocystidia* near the pileus margin extremely rare (only 5 observed), inconspicuous, hardly distinguishable, 58.2–*79.6*–101.0(− 115) × (6–)6.3–*7.0*–7.7(− 8) μm, cylindrical to narrowly clavate, apically obtuse, content as hymenial cystidia but very little, near the pileus centre absent. *Oleiferous hyphae* containing brown pigments present in the trama, *cystidioid hyphae* absent.

*Ecology*: Growing with *Picea abies,* and *Pinus sylvestris*.

*Distribution*: Known from the Czech Republic, Estonia, Finland, Italy, Norway, and the Russian Federation.

*Additional material studied*: **Italy**: Langhestel, with *Picea* and *Pinus sylvestris*, 25 Sept. 1997, *S. Adamčík*, (SAV F-2610). **– Czech Republic**: South Bohemia, Český Krumlov district, Malonty, with *Picea* and *Pinus sylvestris*, together with *Tricholoma matsutake*, 2 Sept. 2014, *J. Borovička*, (PRM 924452).

*Notes*: Our data suggests that *Russula ustulata* has a specific ecology, different from the other species in the complex. It is associated with coniferous trees in boreal forests or mountain habitats.

**Fig. 16:**
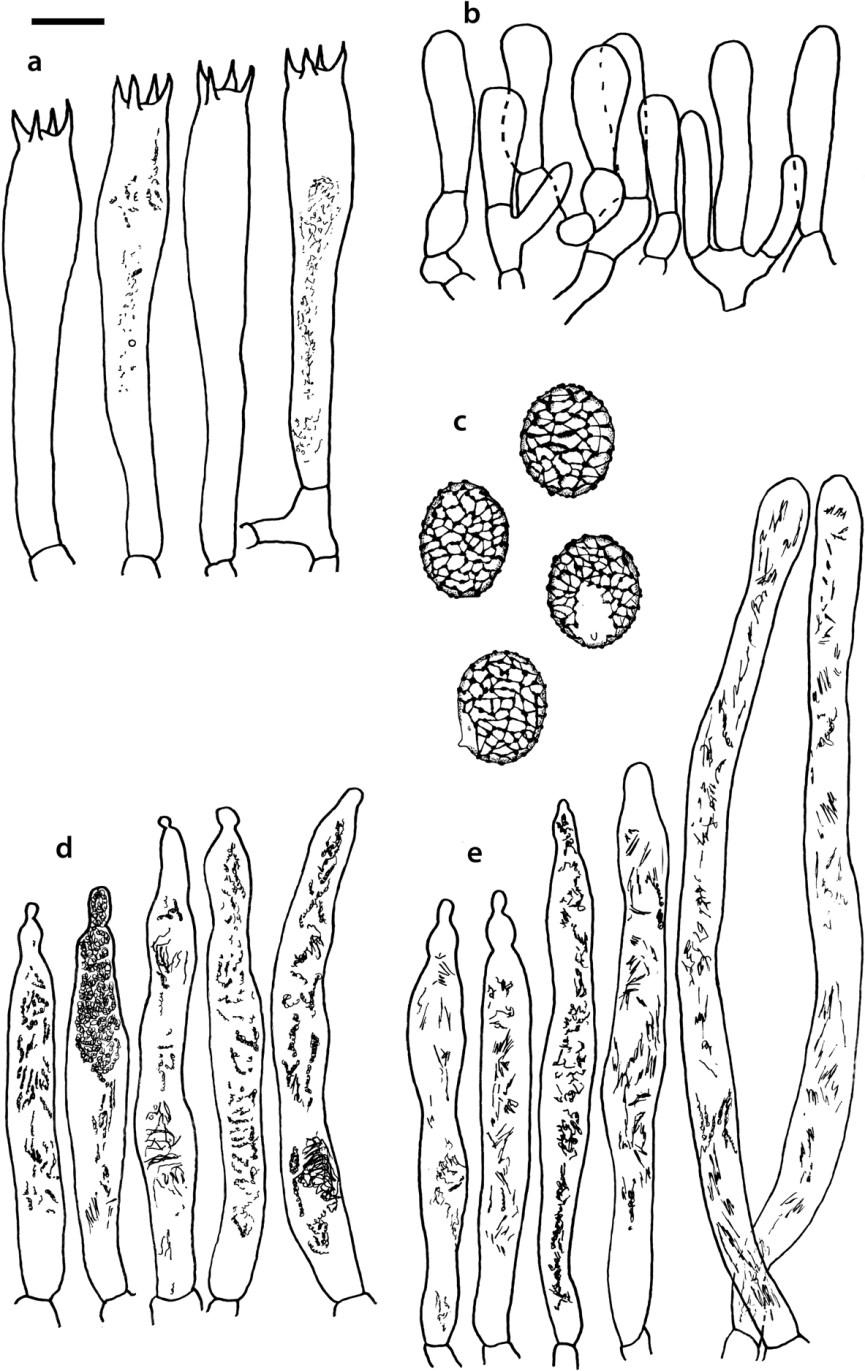
*Russula* sp. 1 (RW 1975), hymenium. **a** Basidia. **b** Marginal cells. **c** Basidiospores. **d** Cystidia near lamellae edges. **e** Cystidia on lamellae sides. Bar = 10 μm, except for c 5 μm

**Fig. 17:**
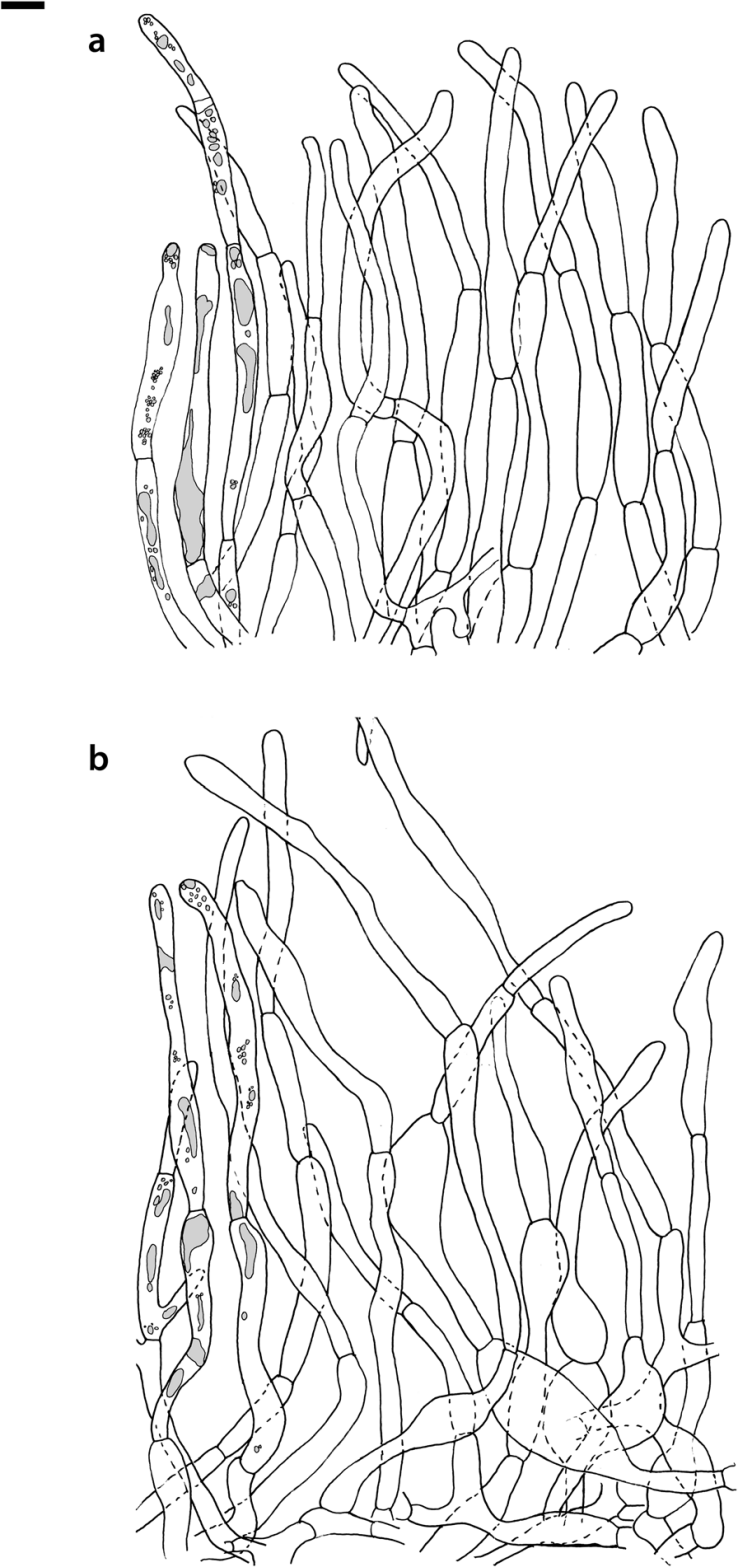
*Russula* sp. 1 (RW 1975), hyphal terminations of the pileipellis. **a** Near the pileus margin. **b** Near the pileus centre. Bar = 10 μm

**Fig. 18:**
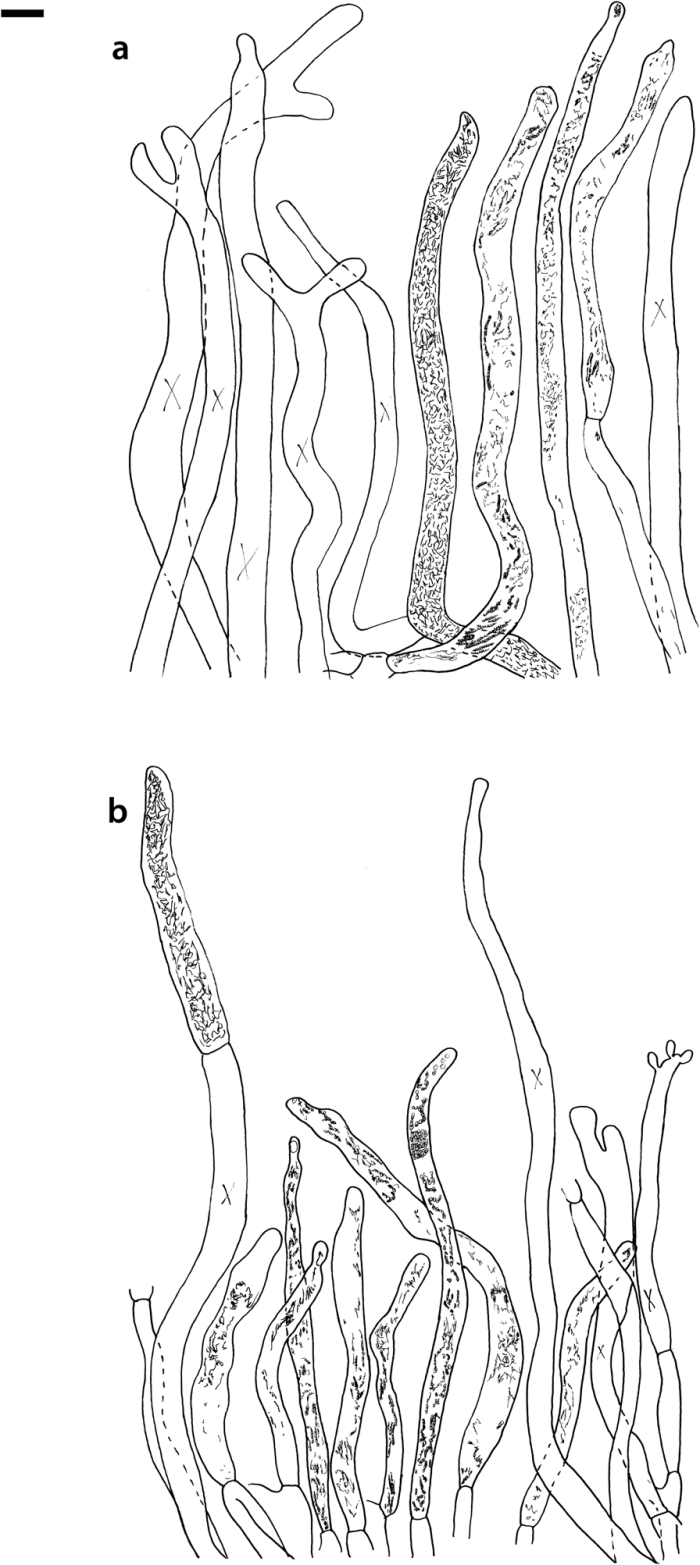
*Russula* sp. 1 (RW 1975), pileocystidia. **a** Near the pileus margin. **b** Near the pileus centre. Bar = 10 μm

***Russula*** **sp. 1** (Figs. 16, 17 and 18).

*Description***:** *Pileus* large, planoconvex to applanate, centrally depressed to umbilicate to infundibuliform; margin straight, smooth; pileus surface smooth, dry, dull; light brown to greyish brown, dark brown, with paler white to sand coloured patches. *Lamellae* narrow, segmentiform to subventricose, adnate to subdecurrent; completely white to yellowish white, quickly blackening; with numerous lamellulae of different lengths; moderately distant; edges even, concolorous, quickly blackening with age. *Stipe* cylindrical, firm and fleshy, smooth to irregular surface, dry; white but turning orange brown to dark grey, black; solid inside. *Context* firm, white, blackening without reddening; taste mild. *Spore print* white (Ia).

*Basidiospores* (7.4–)7.6–*7.9*–8.2(− 8.4) × (6.0–)6.1–*6.3*–6.5(− 6.6) μm, broadly ellipsoid, Q = (1.18–)1.22–*1.26*–1.30(− 1.33); ornamentation of very low, very dense [(8–)9–15(− 16) in a 3 μm diam circle] amyloid warts, to 0.3 μm high, reticulate, abundantly fused into chains [(1–)2–7(− 11) fusions in a 3 μm diam circle], abundantly connected by short, fine line connections [(5–)7–13(− 15) in a 3 μm diam circle]; suprahilar spot medium-sized, not amyloid. *Basidia* (54–)56.7–*60.9*–65.1(− 69) × (9–)9.4–*9.9*–10.4(− 11) μm, narrowly clavate, 4-spored. *Hymenial cystidia*, 64.0–*83.6*–103.2(− 130) × (7–)7.9–*8.7*–9.5(− 10) μm, cylindrical to narrowly fusiform, often slightly moniliform and flexuose, apically obtuse or with double constriction or small appendage, thin-walled; with heteromorphous, oily content, fragmented in multiple crystalline-like masses, without reaction in sulfovanillin; near the lamellae edges, (53–)56.2–*62.3*–68.4(− 76) × (6–)6.9–*7.8*–8.7(− 9) μm, cylindrical to narrowly fusiform, often slightly moniliform and flexuose, apically obtuse or with small appendage, thin-walled; content as on lamellae sides. *Lamellae edges* sterile, elements containing brown pigment when older; *marginal cells* (15–)16.3–*21.5*–26.7(− 30) × (5–)5.3–*6.6*–7.9(− 9) μm, undifferentiated, cylindrical to narrowly clavate, thin-walled. *Pileipellis* orthochromatic in Cresyl Blue, 200–275 μm deep, not sharply delimited from trama and not gradually passing, intermediate; subpellis not delimited from suprapellis; hyphae 3–5 μm wide near trama, more dense near surface and near trama, irregularly oriented, more parallel and horizontal near trama, pigmented only in the upper part of the pileipellis, with some gelatinous coating. *Acid-resistant incrustations* absent. *Hyphal terminations* near the pileus margin long, with multiple septa, scarcely branched at the bases, flexuous, thin-walled, filled with irregular refractive bodies containing brown pigments; terminal cells (39–)47.6–*61.4*–75.2(− 86) × 5.0–*6.0*–7.0(− 8) μm, narrowly cylindrical to slightly subulate or narrowly fusiform, on average apically constricted to 4.5 μm; subterminal cells and the cells below similar in length and width or slightly wider, subterminal cells never branched. Hyphal terminations near the pileus centre slightly wider and apically more attenuated, containing inflated cells, more often branched at the bases; terminal cells (28–)46.3–*70.5*–94.7(− 130) × (3–)3.9–*5.2*–6.5(− 8) μm, subterminal cells rarely branched. *Pileocystidia* near the pileus margin numerous, 1 celled, rarely 2 celled, extremely long, terminal cells 98–more than 320 × (8–)8.2–*9.1*–10.0 μm, cylindrical, flexuose, apically obtuse or with slight constriction, sometimes bifurcating, content as in hymenial cystidia or more granulose, without reaction in sulfovanillin; near the pileus centre numerous, 1–2 celled, generally shorter but still very long, terminal cells 56.6–*117.4*–178.2(− 298) × (5–)6.1–*8.4*–10.7(− 14) μm, similar in shape and content, rarely with up to 3 eccentric appendages. *Oleiferous hyphae* containing brown pigments and *cystidioid hyphae* present in the trama.

*Ecology*: Growing with Mediterranean cork oak (*Quercus suber*).

*Distribution*: Known from Italy (Sardinia).

*Specimen examined*: **Italy**: Sardinia, Tempio Pausania, road SS133, between 3 and 4 km from centre, with *Quercus suber*, 1 Nov. 2000, *R. Walleyn*, RW 1975 (GENT).

*Notes*: Although there is molecular and morphological support that this collection represents a species different from the other species in the *Russula albonigra* complex, the authors chose to not formally describe this species here as the description is based on a single collection only.

### Key to the European species of *Russula* subgen. *Compactae*


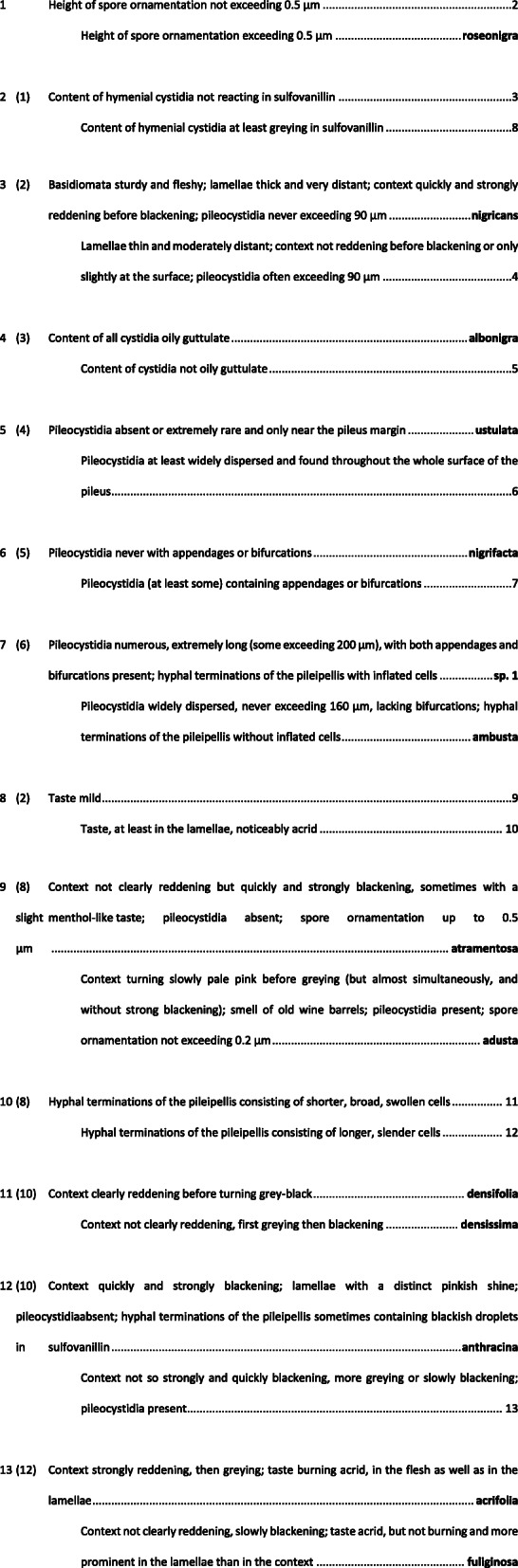


## DISCUSSION

Our study recognises five species within the traditional concept of *Russula albonigra* and this name was the only available at the species rank. Before new names could be assigned to undescribed species, identification of the old name proofed to be challenging, especially because it was already used in the early Friesian period and its original description (Krombholz 1845) does not meet current morphological standards and does not provide sufficient detail. The species clade identified in this study as *R. albonigra* has the best match with the probable geographic and ecological origin of the species described by Krombholz.

The traditional morphological concept of *R. albonigra* (Romagnesi 1967; Sarnari 1998; Kibby 2001) more or less agrees with our observations of the species complex. We think that in the field, for the preliminary identification of species to the *R. albonigra* complex, the following characters are most helpful: a strong and fast blackening of the surface resulting in a distinct contrast of wounded compared to untouched areas, the relatively sturdy and thick-fleshed basidiomata, a mild or refreshing taste of the context, moderately distant and strongly blackening lamellae and a dry, usually dull and not viscid pileus cuticle. However, macroscopically the species within the *R. albonigra* species complex are very alike and cannot be distinguished unambiguously. Nonetheless, we observed that the reaction to FeSO_4_ could be interesting to differentiate some of the species. While *R*. *albonigra* has an orange reaction to FeSO_4_, both *R*. *nigrifacta* and *R*. *ustulata* have a greenish reaction. As this data is missing for *R*. *ambusta* and *R*. sp. 1, it is important to pay attention to this character in future collections. There are some interesting observations questioning the traditional characteristics used to define the *R*. *albonigra* complex. First of all, it seems that the absolute lack of any reddening is not a reliable feature, because it can be weak and easily overseen or vanishing quickly due to the strong and quick blackening. At least three of the species within the *R*. *albonigra* complex (*R*. *albonigra, R*. *nigrifacta,* and *R. ustulata*) comprise a collection where some weak reddening is observed at the surface or even of the context. Some variability about the reddening reaction was also noted by Romagnesi, who recognised *Russula albonigra* f. *pseudonigricans* with an intense reddening context (Romagnesi 1962; Romagnesi 1967). Attempts to get a sequence from the type material failed. The holotype of this form is in a bad condition which does not allow good microscopic observations. Furthermore, a microscopic study of a paratype suggested that holotype and paratype did not represent the same species. We suggest for now, until molecular data becomes available, not to draw any conclusions about the identity of *R*. *albonigra* f. *pseudonigricans* or its classification within the *R*. *albonigra* complex. Moreover, names described in our study have priority at species rank over any future combination of the f. *pseudonigricans* epithet (Art. 11.2 of the ICNafp). Although we believe that the reddening reaction of the context can be used as a diagnostic between species on opposite sides of the spectrum (i.e. species with a strong reddening reaction versus species without a clear reaction) some caution is needed.

Another traditional morphological character to define *R. albonigra* was the characteristic menthol taste in the lamellae. This does not seem to be a stable character because it was not observed in all collections and it can also depend on the subjective opinion of an individual person. Furthermore, this menthol-refreshing taste is also noted to possibly be present in *R*. *atramentosa* by Sarnari (1998).

We found the lack of a reaction of the cystidial content to sulfovanillin to be a good synapomorphic character to define the *R. albonigra* complex. *Russula nigricans* is the only species outside this complex also showing no clear reaction of the cystidial content to sulfovanillin. But the latter species can easily be distinguished from the *R*. *albonigra* complex by its thick and very distant lamellae, the strong reddening of the context and its pileocystidia that are much shorter (never exceeding 90 μm). Our conclusions about the delimitation of the species complex are only based on observations of European taxa of *R*. subgen. *Compactae*.

The second challenge of this study was to define morphological differences among the species of the *R. albonigra* complex defined by phylogenetic analyses. Due to the low morphological variability we could consider the species within the *R*. *albonigra* complex pseudocryptic species (i.e. species with a morphological resemblance that seems indistinguishable at first, but can be distinguished when using the appropriate characters; Delgat et al. 2019). This is a phenomenon that is widely distributed within the *Russulaceae*, especially in the genus *Lactifluus* (Stubbe et al. 2010, Van de Putte et al. 2010, Van de Putte 2012, De Crop et al. 2014, Van de Putte et al. 2016, Delgat et al. 2017, De Lange et al. 2018, Delgat et al. 2019), but also within the genus *Russula* (Adamčík et al. 2016a; Adamčík et al. 2016b; Caboň et al. 2019). The most striking microscopical differences between the species in the *R. albonigra* complex are the higher spore ornamentation of *R*. *nigrifacta* and *R*. *albonigra* compared to the other species within the complex. *Russula ustulata* and *R*. sp. 1 have an almost complete and denser reticulum than the incomplete reticulum of *R*. *nigrifacta* and *R*. *albonigra*. The ornamentation of the spores in *R*. *ambusta* is intermediate in reticulation and density. *R*. *albonigra* is distinguishable by the unique oily guttulate content of all cystidia. *R*. *nigrifacta* typically lacks appendages and bifurcations on the pileocystidia whereas these are present in *R*. *albonigra* and *R*. sp. 1. *Russula ambusta* lacks bifurcations but appendages are present. The most striking feature of *R*. *ustulata* is the absence (or rareness) of pileocystidia, whereas *R*. sp. 1 has very long and numerous pileocystidia. The thickness of the pileipellis is also an interesting character. *R*. *albonigra* has the thinnest pileipellis followed by *R*. *nigrifacta*, the other species in the complex have a much thicker pileipellis. The presence of inflated subterminal cells in the hyphal terminations of the pileipellis centre is typical for *R*. sp. 1 and not observed in the other species of the complex.

Collections used in this study often do not have precise ecological details to define ecological niches and host tree preferences. However, habitat type and geographical data suggest biological relevance to recognise closely related species (Ryberg 2015). *Russula ambusta*, *R. nigrifacta,* and *R. ustulata* are closely related but seem to inhabit ecologically different niches. *Russula nigrifacta* occurs both with Mediterranean oaks (*Quercus ilex* and *Quercus suber*) and *Quercus robur* and *Carpinus betulus* in thermophilous oak forests. Possibly, *Russula* sp. 1 has a similar ecology*,* it is only known from a single collection associated with Mediterranean oak (*Quercus suber*). *Russula ustulata* is up to now only known from boreal or mountain habitats, associated with coniferous trees (*Picea* sp., *Pinus* sp.). *Russula ambusta* and *R*. *albonigra* seem to have a similar ecology and are associated with a variety of trees in temperate to montane forest types. *Russula ambusta* was collected with *Quercus robur*, *Pinus sylvestris,* and *Betula pendula*; *Russula albonigra* with *Fagus sylvatica*, *Abies alba*, *Picea abies,* and *Carpinus betulus*.

Our study suggests that species within the *Nigricantinae* clade have a limited area of distribution, unlike what is often believed. All North American collections retrieved from GenBank and placed in the *R. albonigra* complex are not clustered with the European ones and probably represent different species (Fig. 2). The retrieved ITS data did not confirm that the distribution of *R. albonigra* is not transcontinental (Singer 1958; Hesler 1961; Shaffer 1962; Kibby and Fatto 1990; Thiers 1994), but rather it supports the hypothesis that none of the European taxa within *R*. subgen. *Compactae* are present in the United States (Adamčík and Buyck 2014). The North American collections represent at least two different species with a high macromorphological resemblance to *R*. *albonigra* and they may represent *R. sordida* and *R. subsordida*, both having a weak or negative reaction of the pileocystidia to sulfovanillin (Adamčík and Buyck 2014). This study shows that the *R. albonigra* complex is also represented in Asia by a still undescribed Chinese species.

A multi-locus phylogeny resulting in a strong support of the European species within the *R*. *albonigra* complex, stimulated the detailed search for morphological differences between the species. The species described in this study are defined by integrated taxonomy combining multi-locus molecular data with detailed morphological and ecological data (i.e. distribution, climate, and host data).

Our study demonstrated that all species within the *R*. *albonigra* complex, supported by the strict genealogic concordance and coalescent-based species delimitation, are strictly distinguished at the threshold of 99.5%, that corresponds to a distance of 0.5% when performing a UNITE search. Even a distance of 1% results in only two UNITE species hypotheses both covering multiple phylogenetic species within the complex*.* This shows that there is a low genetic diversity of the ITS region between the species within this complex. A possible explanation for this low genetic diversity is that the species within the *R*. *albonigra* complex are only relatively recently diverged from each other, which could explain the relatively short branch lengths (Fig. 2) and the low morphological variability. The occupation of new habitats and the adaptation to new hosts could have caused the radiation seen.

This study shows that ITS sequence similarity thresholds of 97% commonly used in metabarcoding studies (Pauvert et al. 2019) are not sufficient to differentiate the phylogenetically defined species of the *R*. *albonigra* complex. This observation is also made for other species complexes within the genus *Russula* (Adamčík et al. 2016b). The species thresholds retrieved in this study agree with the conclusions of testing global fungal databases as training datasets, that predicted the optimal identity thresholds to discriminate filamentous fungal species as 99.6% or 99.3% for ITS (Vu et al. 2019; Vu et al. 2020). Badotti et al. (2017) rank *Russula* as the genus with only 38% probability of correct identification, but the study also ranks it into the group for which ITS is a good marker while another *Russulaceae* genus, *Lactarius,* is placed in a group for which ITS is a poor marker. We do not promote the use of a universal threshold value, but rather emphasise the importance of searching for the best threshold value, according to the fungal group of interest.

Our UNITE species hypothesis threshold testing meets both major problems highlighted by Kõljalg et al. (2013): (1) the lack of an inclusive, reliable public reference data set; and (2) the lack of means to refer to fungal species, for which no scientific name is available, in a standardized stable way. First, our singleton collection of *R.* sp*.* 1 is not represented in UNITE, the other four European species are represented by 2–20 sequences. Second, the UNITE nomenclature refers to, in our opinion, an incorrect concept of *R*. *albonigra* and there is a lack of means with which we can assign potential correct and valid available names to the North American taxa.

As the result of a concerted effort to improve UNITE annotations, Nilsson et al. (2014) designated, based on relevant literature data, 1368 species hypothesis reference sequences but also marked 363 sequences of compromised quality. Sequences of compromised technical quality often originate from amplicon sequencing of environmental samples and are biased by the sequencing technique, and some of them can be recognised by automated chimera search or presence of IUPAC ambiguity codes (Badotti et al. 2017; Nilsson et al. 2018). Our comparison of distinguishing nucleotide positions (Additional file 1) demonstrated that short ITS sequences usually do not provide sufficient information for species identification, i.e. sequences UDB0502905 and UDB031025 containing only ITS2 region are undoubtedly identified to species, but UDB0663165 and UDB0557800 provide dubious information. The sequence UDB065518 does not match any species recognised in our tree due to low quality or possibly it represents a new taxon. Our study dealt with five species and we identified four sequences of compromised quality, this is a higher ratio of low quality sequences detected compared to the above mentioned study of Nilsson et al. (2014).

Hofstetter et al. (2019) highlight the main problems with sequence-based identification of fungi. Besides poor taxon coverage in public sequence databases, misidentification, the use of wrong names and bad annotation of sequences, remain a major problem. As sequence-based identification becomes more and more routine and the standard approach for many (mainly ecological) studies, it is clear that the annotation of public sequences urgently needs to be improved. An important implementation is the recent introduction of taxonomic hypothesis that communicate SH with taxonomic identification (Kõljalg et al. 2020). It allows the tracing of taxonomic concepts presented at UNITE and to link them with data about other fungal traits. Hofstetter et al. (2019) and Durkin et al. (2020) state that taxonomy finds itself at the same risk of extinction as the very species they are supposed to study and provide some recommendations towards fungal taxonomists on how to highlight the importance and improve taxonomic work. Taxonomists should create a reliable taxonomic framework that can be used by conservationists and ecologists for sequence based identification. Lucking et al. (2020) provides a discussion on how to improve the quality of identification tools and states that these tools are only as good as the reference data behind them. The UNITE database favours third-party annotation by taxonomic experts to improve the taxonomic annotation of the sequence data (Nilsson et al. 2018). Additional file 3 provides our proposed correction to the annotation of the sequences, with the immediate advantage that it can be directly integrated into further studies.

## CONCLUSION

*Russula albonigra* has always been seen as one of the more easily recognizable species within *R*. subgen. *Compactae* by its strongly and rapidly blackening reaction of the context, without intermediate reddening, resulting in a strong black-and-white contrast of wounded and untouched parts, and the menthol-cooling taste of the lamellae. The species was also thought to cover a broad ecological amplitude and large distribution area. However, molecular analysis revealed that *R*. *albonigra s. lat*. represents a species complex consisting of at least five European, two North American, and one Chinese species. A thorough morphological study shows that the characters traditionally used are not always reliable to define the *R*. *albonigra* complex, instead the lack of a reaction of the cystidial content to sulfovanillin is proposed as a character to improve delimitation of the *R. albonigra* complex. This feature is only shared with *R. nigricans* which can be readily distinguished by the more spaced lamellae. Our UNITE species hypothesis threshold testing revealed perfect phylogenetic species match at a sequence distance threshold of 0.5% for the *R*. *albonigra* complex. UNITE species hypothesis may be a powerful tool to improve knowledge about distribution and ecology of studied species, but the pitfalls include short and low quality sequences. For phylogenetic analyses of the ITS region we recommend the use of sequences with existing (not blank) UNITE species hypotheses at the 0.5% threshold and sequences of full length. Our observations can be applicable for the genus *Russula* as a whole and to many other macrofungal genera. The importance of looking for the best threshold value according to the fungal group of interest is emphasised.

## Supplementary Information

**Additional file 1.** Sequence similarity table. Table showing sequence similarity at distinguishing nucleotide positions.

**Additional file 2.** Comparison table. Table with a detailed comparison of microscopical characters.

**Additional file 3.** Proposed corrected sequence annotation. Table with proposed corrected annotation of the taxonomic data of the public sequences.

## Data Availability

All data generated or analysed during this study are included in this published article [and its supplementary information files].

## Declarations

### Adherence to national and international regulations

We confirm adherence to any pertinent national or international legislation or regulations that apply to the transfer of living biotic materials used in the study between countries.
