# Correction to: Enlightening the black and white: species delimitation and UNITE species hypothesis testing in the *Russula albonigra* species complex

**DOI:** 10.1186/s43008-021-00079-7

**Published:** 2021-10-04

**Authors:** Ruben De Lange, Slavomír Adamčík, Katarína Adamčíkova, Pieter Asselman, Jan Borovička, Lynn Delgat, Felix Hampe, Annemieke Verbeken

**Affiliations:** 1 Research Group Mycology, Department of Biology, Ghent University, K.L. Ledeganckstraat 35, 9000 Ghent, Belgium; 2 Institute of Botany, Plant Science and Biodiversity Center, Slovak Academy of Sciences, Dúbravská cesta 9, 845 23 Bratislava, Slovakia; 3 Institute of Forest Ecology Slovak Academy of Sciences, Akademická 2, 949 01 Nitra, Slovakia; 4 Institute of Geology of the Czech Academy of Sciences, Rozvojová 269, 165 00 Prague 6, Czech Republic; 5 Nuclear Physics Institute of the Czech Academy of Sciences, Hlavní 130, 250 68 Husinec-Řež, Czech Republic; 6 Meise Botanic Garden, Research Department, Nieuwelaan 38, 1860 Meise, Belgium

## Correction to: IMA Fungus (2021) 12:20 https://doi.org/10.1186/s43008-021-00064-0

Following the publication of the original article [1], we were notified of a few inconsistencies between the pdf version and the html version with regards to the Key to the European species of Russula subgen. Compactae (pg. 25).

• In the online version for steps 3(2), 9(8) and 12(10): part of first option was gone and combined with the second option.

•In the online version for step 13(12), both options were present but formed one block of text. ”Context” on the second line should be the start of option two.

The original article has now been corrected.
